# Multi-Channel 4D Parametrized Atlas of Macro- and Microstructural Neonatal Brain Development

**DOI:** 10.3389/fnins.2021.661704

**Published:** 2021-06-16

**Authors:** Alena Uus, Irina Grigorescu, Maximilian Pietsch, Dafnis Batalle, Daan Christiaens, Emer Hughes, Jana Hutter, Lucilio Cordero Grande, Anthony N. Price, Jacques-Donald Tournier, Mary A. Rutherford, Serena J. Counsell, Joseph V. Hajnal, A. David Edwards, Maria Deprez

**Affiliations:** ^1^Department of Biomedical Engineering, School Biomedical Engineering and Imaging Sciences, King's College London, St. Thomas Hospital, London, United Kingdom; ^2^Centre for the Developing Brain, School Biomedical Engineering and Imaging Sciences, King's College London, St. Thomas Hospital, London, United Kingdom; ^3^Department of Forensic and Neurodevelopmental Science, Institute of Psychiatry, Psychology and Neuroscience, King's College London, London, United Kingdom; ^4^Department of Electrical Engineering, ESAT/PSI, KU Leuven, Leuven, Belgium; ^5^Biomedical Image Technologies, ETSI Telecomunicacion, Universidad Politécnica de Madrid, CIBER-BBN, Madrid, Spain

**Keywords:** multi-modal MRI, neonatal brain, spatio-temporal atlas, atlas-based analysis, multi-channel registration, white matter maturation, white matter parcellation

## Abstract

Structural (also known as anatomical) and diffusion MRI provide complimentary anatomical and microstructural characterization of early brain maturation. However, the existing models of the developing brain in time include only either structural or diffusion MRI channels. Furthermore, there is a lack of tools for combined analysis of structural and diffusion MRI in the same reference space. In this work, we propose a methodology to generate a multi-channel (MC) continuous spatio-temporal parametrized atlas of the brain development that combines multiple MRI-derived parameters in the same anatomical space during 37–44 weeks of postmenstrual age range. We co-align structural and diffusion MRI of 170 normal term subjects from the developing Human Connectomme Project using MC registration driven by both T2-weighted and orientation distribution functions channels and fit the Gompertz model to the signals and spatial transformations in time. The resulting atlas consists of 14 spatio-temporal microstructural indices and two parcellation maps delineating white matter tracts and neonatal transient structures. In order to demonstrate applicability of the atlas for quantitative region-specific studies, a comparison analysis of 140 term and 40 preterm subjects scanned at the term-equivalent age is performed using different MRI-derived microstructural indices in the atlas reference space for multiple white matter regions, including the transient compartments. The atlas and software will be available after publication of the article^1^.

## 1. Introduction

In addition to being a routine diagnostic tool in neonatal brain imaging (Rutherford et al., 2010), MRI has been widely used for quantification and interpretation of neonatal brain development in term- and preterm-born infants. Premature birth before 37 weeks postmenstrual age (PMA) is associated with an increased risk of atypical brain maturation leading to neurocognitive and neurobehavioural disorders. Multiple studies demonstrated correlation of MRI metrics with prematurity, clinical and environmental factors and neurodevelopmental outcomes (Ball et al., 2017; Barnett et al., 2018; Dimitrova et al., 2020). In this context, models of normal brain development such as spatio-temporal atlases (Schuh et al., 2018) can also potentially facilitate detection of altered maturation patterns. The advanced acquisition and reconstruction protocols (Cordero-Grande et al., 2018) produce high-resolution structural T1-weighted (T1w) and T2-weighted (T2w) MRI volumes that allow segmentation of fine brain anatomical structures (Makropoulos et al., 2014). But these MRI modalities have low contrast for white matter (WM) structures that also vary during the neonatal stage due to ongoing myelination. On the other hand, lower resolution diffusion MRI reflects the properties of tissue microstructural complexity in terms of diffusivity, anisotropy, neuronal density and fiber orientation (Pannek et al., 2012; Bastiani et al., 2019; Batalle et al., 2019; Feng et al., 2019; Pietsch et al., 2019; Zollei et al., 2019). Combined diffusion and structural MRI analysis has already shown a potential to increase interpretability of brain maturation patterns (Ball et al., 2017).

### 1.1. Structural MRI Metrics

The structural MRI-derived metrics most commonly used in neonatal brain studies include tissue- and structure-specific volumetry (Kuklisova-Murgasova et al., 2011; Makropoulos et al., 2016; Thompson et al., 2019) and surface measurements such as cortical thickness and curvature (Bozek et al., 2018; Fenchel et al., 2020) that can be extracted from automated segmentations (Makropoulos et al., 2014). Recently, automated segmentation of T2w images has also been applied for quantification of the volume of myelinated regions (Wang et al., 2019). Intensity changes in T1w and T2w images characterize white matter injury (O'Muircheartaigh et al., 2020) and diffuse excessive high signal intensity (DESHI) regions (Morel et al., 2021). Quantitative and semi-quantitative metrics applied to developing neonatal brains include the T1w/T2w signal ratio associated with myelin content (Bozek et al., 2018) and T2 relaxometry (Pannek et al., 2013; Kulikova et al., 2015; Wu et al., 2017; Knight et al., 2018).

### 1.2. Diffusion MRI Metrics

Brain microstructure can be probed using a variety of quantitative metrics derived from diffusion MRI. Even though diffusion tensor imaging (DTI) is limited by inconsistencies in fiber-crossing regions (Jeurissen et al., 2013), DTI-derived metrics, including the fractional anisotropy (FA) and the mean, radial and axial diffusitivity (MD, RD and AD) are still most widely used in neonatal brain studies (Barnett et al., 2018; Feng et al., 2019; Thompson et al., 2019; Dimitrova et al., 2020). Recently, higher order metrics, that alleviate some of the limitations of DTI in the fiber crossing regions, have also been applied to investigate neonatal brain development, including the mean kurtosis (MK) index derived from diffusion kurtosis imaging (DKI) (Bastiani et al., 2019) and intracellular volume fraction (ICVF), fiber orientation dispersion index (ODI) and volume fraction of the isotropic compartment (FISO) derived from Neurite Orientation Dispersion and Density Imaging (NODDI) model (Zhang et al., 2012). The NODDI-derived indices have been used to characterize development of both white and gray matter microstructural features (Kunz et al., 2014; Batalle et al., 2019; Fenchel et al., 2020; Kimpton et al., 2020). The microscopic fractional anisotropy (μFA) index (Kaden et al., 2016) designed to disentangle microscopic diffusion anisotropy from the orientation dispersion has not yet been applied to neonatal brains. Constrained spherical deconvolution (CSD) (Tournier et al., 2007; Jeurissen et al., 2014) allows extraction of orientation-resolved microstructural information as orientation distribution functions (ODF) from multi-shell high angular resolution diffusion imaging (HARDI) data. Based on fiber ODF, fixel-based analysis (Raffelt et al., 2017) provides the means for assessment of specific fiber populations in terms of fiber density (FD) and fiber-bundle cross-section (FC) (Pannek et al., 2018; Pecheva et al., 2019).

### 1.3. Atlases and Models of Neonatal Brain Development

Spatio-temporal normalization and construction of age-specific group-average templates have been routinely employed in processing pipelines in the recent large neonatal brain MRI studies to detect inter-group differences and anomalies in individual brains (Oishi et al., 2019). The majority of the reported spatio-temporal population-averaged atlases of the neonatal brain include either structural (T2w and T1w) (Kuklisova-Murgasova et al., 2011; Serag et al., 2012; Schuh et al., 2014, 2018; Wright et al., 2014; Makropoulos et al., 2016; Schwartz et al., 2016; Wang et al., 2019; O'Muircheartaigh et al., 2020) or diffusion (Feng et al., 2019; Pietsch et al., 2019; Dimitrova et al., 2020) channels. In this context, the term channel means an image of a single MRI contrast that is a part of a group of images belonging to the same subject. To our knowledge, the only existing multi-channel population-averaged 3D T1w+T2w+DTI atlas (Oishi et al., 2011) was constructed from a set of normal term subjects from 38 to 41 weeks PMA. However, the averaged template was reported to have significantly lower sharpness than the original T2w and DTI images. Apart from Feng et al. (2019) and Pietsch et al. (2019) who used FA+MD or multi-component ODF channels for registration, these atlases were constructed based on registration driven by a single channel and the output transformations were propagated to the rest. The reported multi-channel (MC) registration methods for brain studies are based on either combination of FA+structural (Park et al., 2003; Forsberg et al., 2011; Geng et al., 2012; Roura et al., 2015) or DTI+structural channels (Avants et al., 2007; Gupta et al., 2015; Irfanoglu et al., 2016). However, DTI-extracted metrics are characterized by inconsistencies in fiber-crossing regions (Tournier et al., 2012). In general, one of the challenges of multi-channel registration is considered to be the alignment between the structural and diffusion MRI volumes. Following spatial normalization, the templates are generally created using either weighted or direct averaging of the signal in the reference space. As an alternative, (Zhang et al., 2016) proposed to perform averaging in the frequency domain and reported higher sharpness of the atlas features.

Due to rapid changes of structure, volume and cytoarchitecture during the fetal and neonatal period, the majority of the atlases have also been resolved in time in the form of weekly templates. Smooth transitions between the atlas time points have been provided through kernel regression (Kuklisova-Murgasova et al., 2011; Serag et al., 2012; Schuh et al., 2014, 2018), logistic regression (Wang et al., 2019) or Gaussian process regression (Marquand et al., 2016; Dimitrova et al., 2020; O'Muircheartaigh et al., 2020). Recently, a Gompertz function (GF) was successfully used to parametrize fetal and neonatal brain volumetry and surface measurements (Wright et al., 2014; Makropoulos et al., 2016; Schwartz et al., 2016), showing better approximation than the linear model (Makropoulos et al., 2016), even though the changes in averaged structural (O'Muircheartaigh et al., 2020) and DTI (Bastiani et al., 2019; Feng et al., 2019; Dimitrova et al., 2020) metrics in white and gray matter can be approximated by linear trends. However, so far, there has been no reported works combining structural and diffusion MRI into a spatio-temporal atlas of the normal term born neonatal brain development.

### 1.4. Region Specific Analysis

The majority of neonatal brain studies have employed region-specific quantitative analyses based on correlation between the MRI-derived metrics measured within specific regions and parameters such as gestational age (GA) at birth, clinical factors or neurodevelopmental outcomes. In structural-only MRI datasets, segmentation is normally performed by atlas-based methods (Makropoulos et al., 2014). In the WM atlas-based analysis, the parcellation maps for the single-subject or population-average WM DTI atlases (Oishi et al., 2011; Feng et al., 2019; Alexander et al., 2020) were created by 2D manual delineation based on DTI directionally-encoded color maps for single subject or population-averaged templates. Label propagation based on DTI channel-guided registration has been widely used in neonatal brain studies (Kersbergen et al., 2014; Rose et al., 2014; Wu et al., 2017; Claessens et al., 2019; Feng et al., 2019). The tract-based spatial statistics (TBSS) (Smith et al., 2006) approach uses skeletonized FA maps for definition of the regions (Krishnan et al., 2016; Barnett et al., 2018; Young et al., 2018; Thompson et al., 2019). As an alternative, tract-specific analysis employs tractography to identify and segment the major WM pathways (Kulikova et al., 2015; Akazawa et al., 2016; Pecheva et al., 2017; Bastiani et al., 2019; Zollei et al., 2019; Dubner et al., 2020; Kimpton et al., 2020). In this case, the seed regions for tractography are defined in the template space and the segmentation of WM tracts is achieved by thresholding of the resulting probabilistic tractography maps. In Akazawa et al. (2016), this approach was also used to create population-specific average probabilistic maps of the major WM tracts.

### 1.5. Contributions

In this work, we propose to merge multiple metrics extracted from both diffusion and structural MRI in a single multi-channel spatio-temporal atlas of normal neonatal brain development parametrized using Gompertz function.

The generated 4D multi-channel atlas covers 37 to 44 weeks PMA range and includes structural (T1w, T2w and T1w/T2w myelin contrast) and diffusion channels with ODF, DTI, DKI, μFA and NODDI derived metrics. Furthermore, the atlas includes two parcellation maps: (i) the major WM tract regions (Alexander et al., 2020) refined using probabilistic tractography in the template space and (ii) a map of the transient WM regions associated with high maturation rates during the neonatal period. To ensure accuracy of spatial alignment, we propose MC registration method (Uus et al., 2020) guided by spatially-weighted structural MRI, diffusion (ODF) MRI and cortical segmentation (Makropoulos et al., 2018) channels. Parametrization in time is performed by the Gompertz function widely used for fitting of growth data. We implemented the atlas construction and fitting functionalities based on the MRtrix3 software package (Tournier et al., 2019). To demonstrate the application of the proposed atlas we perform a multi-modality study to compare term and preterm brain development and identify regions where WM maturation has been altered by preterm birth.

## 2. Materials and Methods

### 2.1. Cohort, Datasets and Preprocessing

**Figure 1:**
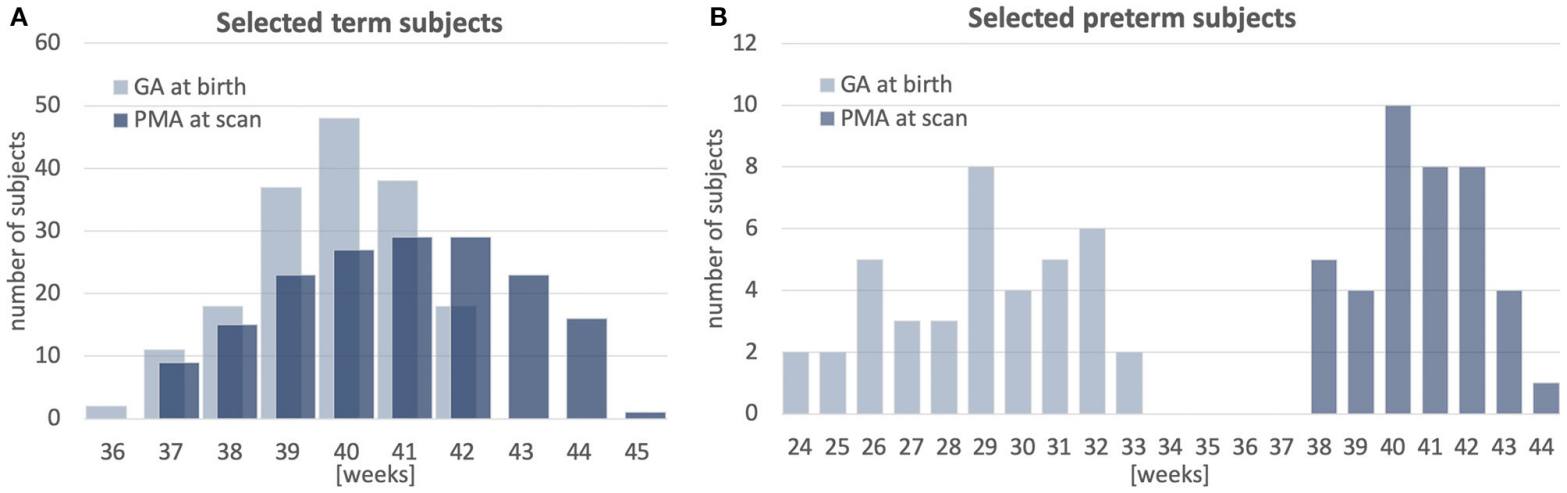
Selected cohort of neonatal subjects from dHCP project: GA at birth and PMA at scan of 170 term subjects **(A)** and 40 preterm subjects **(B)**.

The atlas was constructed using 170 multi-modal MRI datasets of term-born neonates (born and scanned between 37 and 44 weeks PMA) that included T1w, T2w and HARDI scans. An additional 40 datasets of preterm neonates (born between 23 and 32 weeks GA: 28.94∓2.54 and scanned between 37 and 44 weeks PMA) were used for comparison analysis. Inclusion criteria were high image quality for scans of all modalities, singleton pregnancies and no major brain abnormalities. All scans were acquired under the developing Human Connectome Project (dHCP)^2^. The datasets were qualitatively assessed and graded by a team of dCHP researchers in terms of the reconstruction and motion correction quality, SNR levels, presence of artifacts and the global coverage of the brain ROI. Only the datasets with the best image quality were selected for this particular study. The distribution of the GA at birth and PMA at scan is given in Figure 1.

The datasets were acquired without sedation on a 3T Philips Achieva scanner equipped with a dedicated 32-channel neonatal head coil and baby transportation system (Hughes et al., 2017). The multi-shell HARDI volumes were acquired with four phase-encode directions on four shells with *b*-values of 0(20), 400(64), 1000(88) and 2, 600(128)*s*/*mm*^2^, TE 90*ms*, TR 3800*ms* (Hutter et al., 2018; Tournier et al., 2020) with 1.5 × 1.5 × 3*mm* resolution and 1.5*mm* slice overlap and reconstructed to 1.5*mm* isotropic resolution using the spherical harmonics and radial decomposition (SHARD) pipeline (Christiaens et al., 2018, 2021) that includes slice-wise motion correction, distortion correction and exclusion of corrupted slices. Prior to reconstruction, the diffusion datasets were preprocessed using the dedicated dHCP pipeline including: Marchenko-Pastur-PCA-based denoising (Veraart et al., 2016) (MRtrix3^3^), Gibbs ringing removal (Kellner et al., 2016), susceptibility and eddy-current distortion correction and inter-volume motion correction with outlier replacement using topup (Andersson et al., 2003) (FSL^4^) and eddy (Andersson and Sotiropoulos, 2016) (FSL), bias field correction based on the *b* = 0 shell using N4 (Tustison et al., 2010) (ANTs^5^).

The structural T2w volumes were acquired using a TSE sequence with TR 12*s*, TE 156*ms*. The T1w volumes were acquired using an IR TSE sequence with TR 4.8*s*, TE 8.7*ms*. The isotropic T2w and T1w volumes with 0.5*mm* resolution were reconstructed using a combination of motion correction (Cordero-Grande et al., 2018) and super-resolution reconstruction (Kuklisova-Murgasova et al., 2012). Intensities of individual T1w and T2w volumes were bias-corrected and normalized to the same intensity ranges as a part of the standard dHCP preprocessing pipeline based on DRAW-Em^6^ (Makropoulos et al., 2014, 2018). In addition, the T2w images were normalized with respect to mean CSF signal intensity. The brain tissue and structure segmentations were generated by DRAW-Em pipeline (Makropoulos et al., 2014). For each dataset, the structural and diffusion volumes were coaligned based on affine registration of T2w and MD volumes using normalized cross-correlation (NCC) similarity metric implemented in MRTrix3. The diffusion-weighted imaging (DWI) volumes were globally normalized prior to the nonlinear multi-channel registration step (Tournier et al., 2019).

### 2.2. Extraction of MRI Metrics

The structural metrics include normalized T1w and T2w intensities and the T1w/T2w ratio reported to be associated with the myelin content (Glasser and Van Essen, 2011). Furthermore, we extracted Jacobians (J) of deformation fields from the MC registration output (section 2.4) to measure local volumetric changes.

The DTI metrics included MD, RD and FA extracted using MRtrix3 toolbox (Tournier et al., 2019). The DKI fitting and calculation of MK was performed similarly to Bastiani et al. (2019). The NODDI (Zhang et al., 2012) toolbox was used for fitting FISO, ICVF and ODI metrics. The estimation of micro FA maps was performed using SMT toolbox (Kaden et al., 2016). Only the two top HARDI shells were used for μFA and DKI fitting in order to minimize the impact of artifacts. In addition, we computed the mean DWI signal mDWI for the top 2, 600*s*/*mm*^2^ shell since it provides high contrast for WM structures. We extracted WM ODF from HARDI using MRtrix3 multi-shell multi-tissue constrained spherical deconvolution (Jeurissen et al., 2014). The track density imaging (TDI) maps were generated in the original space of dMRI volumes from the outputs of the standard MRtrix3 probabilistic tractography based on the 2nd order integration over fiber orientation distributions (iFOD2) (Tournier et al., 2010, 2019) with whole brain as the seed region and 700,000 streamlines for all datasets. This particular number of streamlines was selected arbitrarily.

### 2.3. Multi-Channel Registration of Blue Combined Structural and HARDI MRI Datasets

We propose a multi-channel non-linear registration technique to improve accuracy of spatial normalization of both structural and diffusion MRI images. The method is build on a multi-contrast ODF registration framework (Raffelt et al., 2011; Pietsch et al., 2017) implemented in MRtrix3 (Tournier et al., 2019) which employs SyN Demons (Avants et al., 2007) with an SSD metric and reorientation of ODF using apodized point spread functions (Raffelt et al., 2012). In order to decrease the sensitivity to acquisition or physiology related changes in signal intensities, we propose to replace the the standard SSD metric with a new robust local angular correlation (LAC) registration metric for ODF channels, which is an extension of angular correlation (Anderson, 2005) originally proposed for for quantitative assessment of ODF datasets. We further add structural and tissue parcellation channels with local NCC (LNCC) similarity measure. The channels are combined through weighted fusion of the displacement field updates (Forsberg et al., 2011). Implementation of the LAC and LNCC metrics is based on the registration pipeline in MRtrix3 (Tournier et al., 2019) that includes reorientation of ODF (Raffelt et al., 2012).

In ODF diffusion model, diffusion signal is represented as a linear combination of real valued spherical harmonic (SH) orthonormal basis functions *Y*_*lm*_(θ, ϕ). For the task of image registration, two dMRI volumes can be expressed in terms of spatially varying spherical functions *A*^*ODF*^(θ, ϕ, *x*) and *B*^*ODF*^(θ, ϕ, *x*), where θ, ϕ are coordinates on the sphere and *x* is a spatial location:

(1) $$\begin{array}{r} A^{ODF}\left(\theta ,\phi ,x\right)=\overset{\propto }{\underset{l=0}{\sum }}\overset{l}{\underset{m=-l}{\sum }}a_{lm}\left(x\right)Y_{lm}\left(\theta ,\phi \right)\\ B^{ODF}\left(\theta ,\phi ,x\right)=\overset{\propto }{\underset{l=0}{\sum }}\overset{l}{\underset{m=-l}{\sum }}b_{lm}\left(x\right)Y_{lm}\left(\theta ,\phi \right) \end{array}$$

We define **local angular correlation** *r*_*a*_ between *A*^*ODF*^ and *B*^*ODF*^ as:

(2) $${\text{ r}}_{a}\left(x\right)\text{ =}\frac{{\langle A,B\rangle }_{x}}{{\langle A\rangle }_{x}^{\frac{1}{2}}{\langle B\rangle }_{x}^{\frac{1}{2}}}=\frac{\sum_{x^{\prime }\in N(x)}\sum_{l=2}^{L}\sum_{m=-l}^{l}a_{lm}(x^{\prime })b_{lm}(x^{\prime })}{{\left(\sum_{x^{\prime }\in N(x)}\sum_{l=2}^{L}\sum_{m=-l}^{l}a_{lm}^{2}(x^{\prime })\right)}^{\frac{1}{2}}\left(\sum_{x^{\prime }\in N(x)}\sum_{l=2}^{L}\sum_{m=-l}^{l}b_{lm}^{2}(x^{\prime })\right){}^{{}^{\frac{1}{2}}}},$$

where *A* and *B* are 4D images of SH coefficients of order *L* with even *l* = {2, 4, ..., *L*} harmonic degree terms, e.g., *A*(*x*) = {_*a*_*lm*_(*x*)}*l* = 2, ..., *L, m* = −*l*, ..., *l*_ and *B*(*x*) = {_*b*_*lm*_(*x*)}*l* = 2, ..., *L, m* = −*l*, ..., *l*_, *N*(*x*) is the local neighborhood centered at *x*, and < >_*x*_ denotes the inner product calculated over *N*(*x*). *A*(*x*) and *B*(*x*) are also normalized with respect to local means (Avants et al., 2008). In this case, the *l* = 0 term does not contribute to *r*_*a*_ values.

Since this is a correlation metric, the corresponding symmetric updates to the displacement fields Λ^*A*^ and Λ^*B*^ can be computed in a similar manner to LNCC demons (Avants et al., 2008):

(3) $$\begin{array}{r} \Lambda^{A}\left(x\right)=\frac{2{\langle A,B\rangle }_{x}}{{\langle A\rangle }_{x}{\langle B\rangle }_{x}}\left(B\left(x\right)-\frac{{\langle A,B\rangle }_{x}}{{\langle A\rangle }_{x}}A\left(x\right)\right)\nabla A\left(x\right) \end{array}$$

$$\begin{array}{l} \Lambda^{B}\left(x\right)=\frac{2{\langle A,B\rangle }_{x}}{{\langle A\rangle }_{x}{\langle B\rangle }_{x}}\left(A\left(x\right)-\frac{{\langle A,B\rangle }_{x}}{{\langle B\rangle }_{x}}B\left(x\right)\right)\nabla B\left(x\right) \end{array}$$

Note that LAC operates in 4D (3D space plus SH dimension) while LNCC is calculated in 3D spatial neighborhood for each individual ODF channel separately (Raffelt et al., 2011).

In the proposed multi-channel registration pipeline, the fixed and moving inputs consist of a set of structural (e.g., T2w) and ODF channels *i* = 1, ..., *I*. At every iteration, the fixed *A*_*i*_ and moving *B*_*i*_ images are registered individually resulting in $\Lambda_{i}^{A}$ and $\Lambda_{i}^{B}$ updates to the displacement fields. The contributions from each of the channels to the global symmetric displacement field updates $\Lambda_{MC}^{A}$ and $\Lambda_{MC}^{B}$ are locally weighted by 3D gradient certainty maps based on the approach proposed in Forsberg et al. (2011).

First, at every iteration, the certainty gradient maps $\alpha_{i}^{A}$ and $\alpha_{i}^{B}$ are computed from the current version of warped channels *A*_*i*_ and *B*_*i*_ (including both structural and ODF volumes) and normalized as:

(4) $$\begin{array}{r} \alpha_{i}^{A}=∥\nabla A_{i}^{T}\nabla A_{i}∥,\;{\hat{\alpha_{i}}}^{A}=\frac{\alpha_{i}^{A}}{max\left(\alpha_{i}^{A}\right)} \end{array}$$

Then, the global symmetric MC updates to the displacement fields $\Lambda_{MC}^{A}$ and $\Lambda_{MC}^{B}$ are computed by weighted averaging of the channel-specific update fields

(5) $$\begin{array}{r} \Lambda_{MC}^{A}=\frac{\sum_{i}{\hat{\alpha_{i}}}^{A}\Lambda_{i}^{A}}{\sum_{i}{\hat{\alpha_{i}}}^{A}},\;\Lambda_{MC}^{B}=\frac{\sum_{i}{\hat{\alpha_{i}}}^{B}\Lambda_{i}^{B}}{\sum_{i}{\hat{\alpha_{i}}}^{B}} \end{array}$$

**Figure 2:**
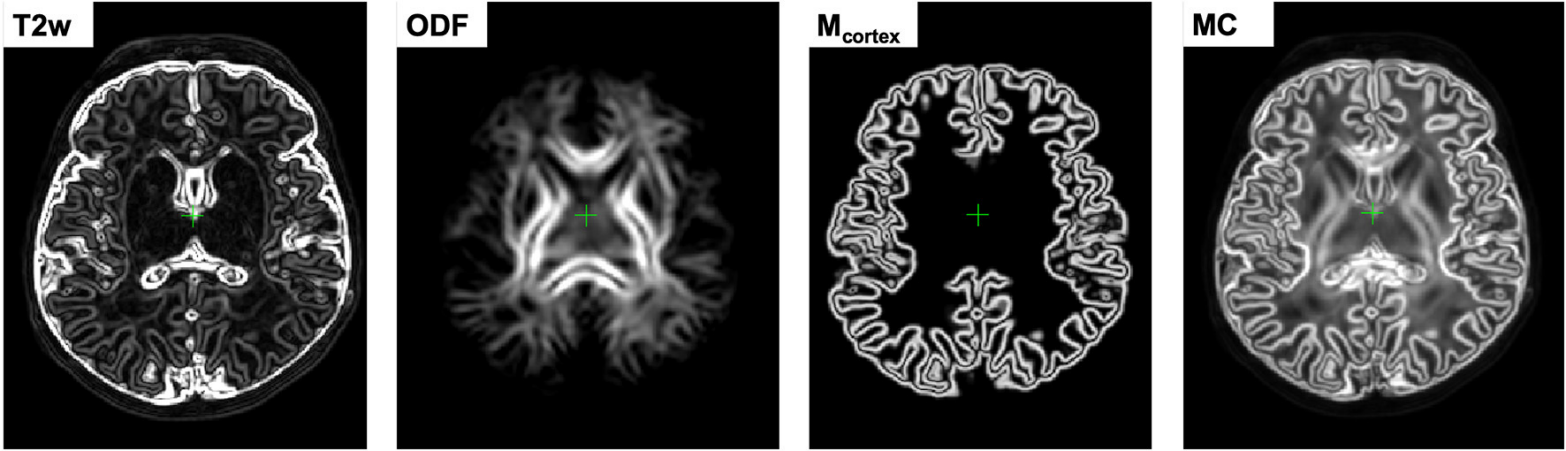
An example of gradient-based certainty maps of T2w, ODF and cortex mask channels computed for one of the dHCP subjects along with the average MC gradient map used for normalization.

This downweights the contributions of the regions in individual channels characterized by low contrast, ensuring that the output deformation fields are locally defined by the channels with the highest structural content. In comparison, the multi-channel SyN approach (Avants et al., 2007) or the existing alternative DTI-based MC registration methods (Geng et al., 2012; Gupta et al., 2015) employ simple averaging of the individual channel updates. Figure 2 shows an example of certainty maps of T2w, ODF and cortex mask channels computed for one of the dHCP subjects along with the average MC weights used for normalization.

### 2.4. Generation of 4D Multi-Channel Atlas

**Figure 3:**
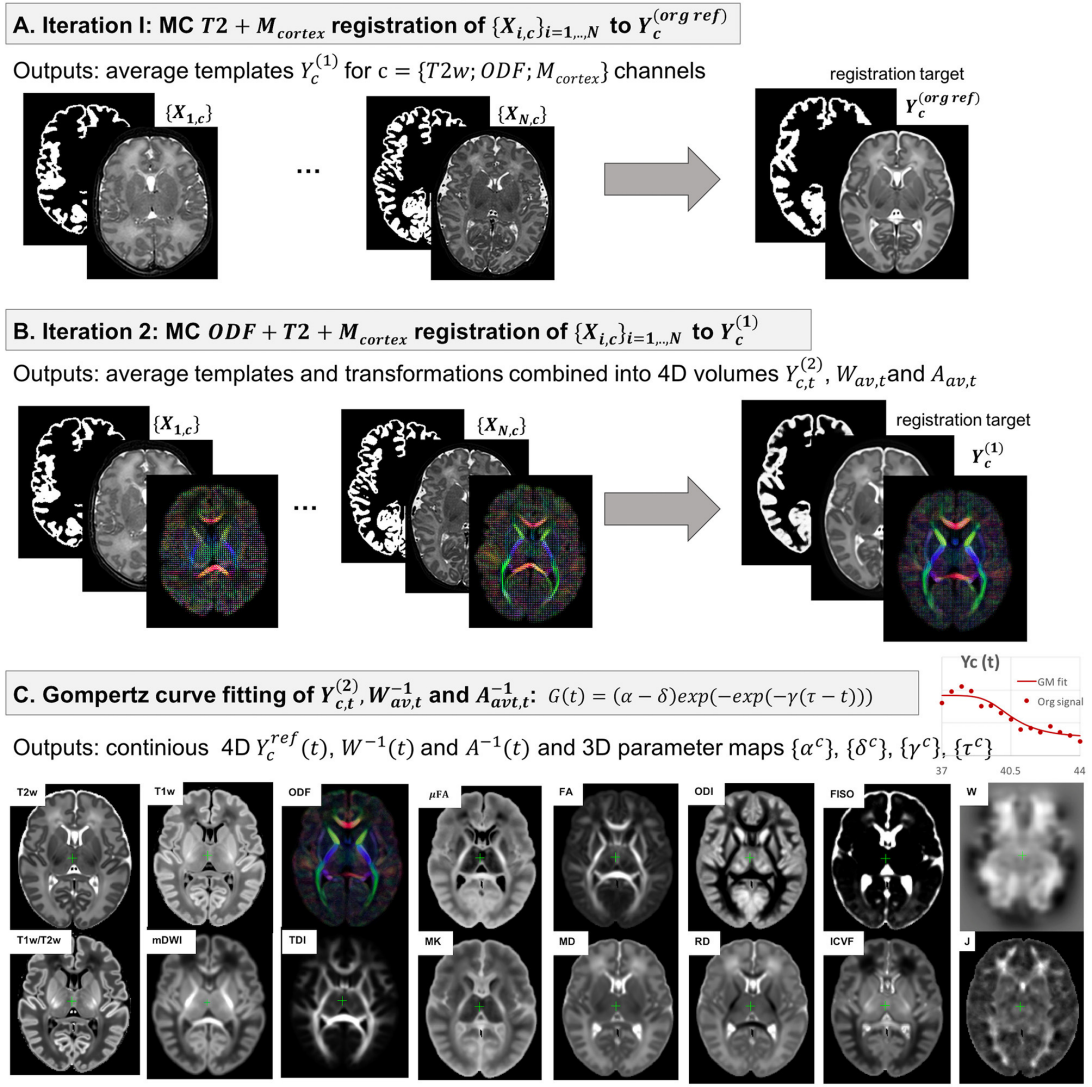
The proposed pipeline for generation of parametrized 4D MC atlas of neonatal brain development during 37–44 weeks PMA range.

The 4D parametrized MC atlas of neonatal brain development was generated from 170 term neonatal datasets in three sequential steps: (A) initial registration of structural channels to a single structural template and creation of an average multi-channel template, (B) refined registration of structural and diffusion channels to the multi-channel template and creation of age-dependent average multi-channel templates, (C) fitting of the signal and deformation fields in time using the Gompertz function to generate the parametrized 4D multi-channel atlas. The proposed pipeline is summarized in Figure 3.

#### 2.4.1. Generation of a 3D Multi-Channel Template

We chose the T2w 36 week template from the dHCP neonatal brain atlas^7^ (Schuh et al., 2018) as the global 3D reference space (*Y*^(*reforg*)^) due to the lower degree of gyrification that facilitates more accurate registration of the cortex. All datasets {*X*_*i*_},_*i* = 1, ..., *N*_ were registered to this template using affine alignment with global NCC followed by non-linear registration guided by two structural channels (T2w + cortex mask), similarly to O'Muircheartaigh et al. (2020):

(6) $$\begin{array}{r} W_{i}^{\left(1\right)}={𝔇}^{LNCC}\left(Y_{c}^{\left(reforg\right)},X_{i,c}\right),{\;}_{c=\left\{T2;\;M_{cortex}\right\};i=1,...,N}, \end{array}$$

where 𝔇 is the MC Demons registration operator, $W_{i}^{\left(1\right)}$ are the output deformation warps for each of the *N* datasets *X*_*i, c*_ with *c* = {*T*2; *M*_*cortex*_} channels and $Y_{c}^{\left(ref\right)}$ is the reference volume. The MC registration included spatially weighted fusion of the channels (section 2.3, Uus et al., 2020). The output deformation warps $\left\{W_{i}^{\left(1\right)}\right\}{,}_{i=1,...,N}$ were propagated to the rest of the structural and dMRI channels. The preliminary set of 3D MC templates $\left\{Y_{c}^{\left(1\right)}\right\}{,}_{c=\left\{T2;\;M_{cortex};\;normODF\right\}}$ was generated by weighted averaging of all registered volumes of T2w, cortex mask and normalized (section 2.1) ODF channels (Figure 3A).

#### 2.4.2. Generation of Age-Specific Multi-Channel Templates

At the second iteration (Figure 3B), we used registration with T2w + cortex mask + normalized ODF channels (section 2.3) to align all datasets to the multi-channel template (section 2.4.1):

(7) $$\begin{array}{r} W_{i}^{\left(2\right)}={𝔇}^{LNCC+LAC}\left(Y_{c}^{\left(1\right)},X_{i,c}\right),{\;}_{c=\left\{T2;\;M_{cortex};\;normODF\right\};i=1,...,N} \end{array}$$

Next, the datasets were divided into 15 subsets according to PMA, to sample the range from 37 to 44 weeks PMA into 0.5 week time-windows. Each of the subsets *N*^*t*^ contains 6-17 subjects depending on availability. The templates $Y_{c,t}^{\left(2\right)}$ for each of the metrics (*c*) described in section 2.2 were generated by robust weighted averaging of the metric maps *X*_*i, c*_ transformed with $W_{i}^{\left(2\right)}$ in subsets *i*∈*N*^*t*^:

(8) $$\begin{array}{r} Y_{c,t}^{\left(2\right)}=\underset{i\in N^{t}}{\sum }\omega_{i,c}\cdot \Theta \left(X_{i,c},W_{i}^{\left(2\right)}\right)/\underset{i\in N^{t}}{\sum }\omega_{i,c},{\;}_{t=37,...,44}, \end{array}$$

where Θ is the transformation operator, *c* is the list of all channels (see Figure 3C). The voxel-wise weights ω_*i, c*_ are binary maps with all values with >1.5 standard deviations from the mean set to zero. This minimizes the impact of outliers due to any abnormalities, artifacts or local misregistrations are excluded.

The templates $Y_{c,t}^{\left(2\right)}$ are biased toward 36 weeks reference space, therefore we calculate the transformations to remove this bias for each time-point. Since the registration is symmetric, it is acceptable to choose the inverse warps ${\left(W_{i}^{\left(2\right)}\right)}^{-1}$ to create the transformation $W_{av,t}^{-1}$ from the age-specific average space to the global reference space:

(9) $$\begin{array}{r} W_{av,t}^{-1}=\underset{i\in N^{t}}{\sum }{\left(W_{i}^{\left(2\right)}\right)}^{-1}/N^{t},{\;}_{t=37,...,44} \end{array}$$

Similarly, we create average inverse affine transformation $A_{av,t}^{-1}$ by selecting only the scaling and shearing components, followed by averaging and inverting.

#### 2.4.3. Parametrized 4D Multi-Channel Atlas

In the final step, a continuous 4D spatio-temporal multi-channel model of the developing neonatal brain (Figure 3C) was constructed by fitting the Gompertz growth curves to the time-dependent average metric maps and transformations. We propose the following form of the Gompertz function since it allows interpretation of both growth rate (γ) and peak in time (τ):

(10) $$\begin{array}{r} G\left(t\right)=\left(\alpha -\delta \right)exp\left(-exp\left(-\gamma \left(\tau -t\right)\right)\right)+\delta , \end{array}$$

where *t* is the time point, α and δ control the upper and lower limits of *G*(*t*), γ represents the growth rate and τ is the center point corresponding to the growth peak. The model was fitted to the time-dependent average metric maps $Y_{c,t}^{\left(2\right)}$ and transformations $W_{av,t}^{-1},A_{av,t}^{-1}$ using least square minimization to produce continuous spatio-temporal maps in the reference space as well as average inverse transformations:

(11) $$\begin{array}{r} Y_{c}^{ref}\left(t\right)=G\left(\alpha^{c},\delta^{c},\gamma^{c},\tau^{c},t\right),{\;}_{t=\left[37;44\right]} \end{array}$$

(12) $$\begin{array}{r} W^{-1}\left(t\right)=G\left(\alpha^{W},\delta^{W},\gamma^{W},\tau^{W},t\right),{\;}_{t=\left[37;44\right]} \end{array}$$

(13) $$\begin{array}{r} A^{-1}\left(t\right)=G\left(\alpha^{A},\delta^{A},\gamma^{A},\tau^{A},t\right),{\;}_{t=\left[37;44\right]}, \end{array}$$

where α^*c*^, δ^*c*^, γ^*c*^ and τ^*c*^ are the Gompertz function parameters of metrics c = {T1w; T2w; T1w/T2w; mDWI; ODF: SH ODF, TDI; DTI: MD, RD, FA; DKI: MK; NODDI: ODI, FISO, ICVF; μFA; Jacobian} and *t* is continuous over 37–44 weeks PMA range. Unbiased spatio-temporal maps *Y*_*c*_(*t*) are obtained by applying nonlinear transformation *W*^−1^(*t*) followed by affine transformation *A*^−1^(*t*) to the biased spatio-temporal maps $Y_{c}^{ref}\left(t\right)$.

### 2.5. Parcellation of WM Regions

The dHCP structural atlas (Schuh et al., 2018) already provides parcellations of cortical and subcortical regions based on DRAW-EM pipeline (Makropoulos et al., 2014), therefore, this work specifically focuses on WM tracts and transient regions. At first, we propagated the parcellation map of the major WM tract regions from M-CRIB-WM atlas (a single subject template at 41 weeks PMA Alexander et al., 2020) by registration of one of the T2w M-CRIB-WM atlas subjects to our T2w 44 week template $Y_{T2w}^{ref}\left(44\right)$.

Then we performed the MRTrix3 iFOD2 probabilistic tractography (Tournier et al., 2010) in $Y_{ODF}^{ref}\left(41\right)$ channel for each of the 54 WM regions (defined in Alexander et al., 2020) with propagated labels as seeds. We performed the tractography in the average template because of the lower noise levels due to averaging. This was followed by manual refinement of all labels using the 3D brush with thresholding editing tool in 3DSlicer (Fedorov et al., 2012) based on the thresholded TDI maps for individual tracts and inspection of the FA and T2 channels. The procedure was performed in three iterations with iFOD2 tractography being performed for the WM ROIs refined in the previous step. The labels were created in the atlas reference space resampled to 0.5*mm* isotropic resolution to account for finer WM structures.

The transient WM regions were localized as regions with high rates of signal changes during 37–44 weeks PMA. The parcellation was generated semi-automatically from the γ^*av*^ map obtained by averaging the absolute of growth rate γ^*c*^ maps for T1w, T2w, RD and FISO channels. These channels were selected since they showed similar patterns in the region associated with the transient fetal compartments (Pittet et al., 2019). The γ^*av*^ map (with values varying within [0;0.5]) was thresholded at 0.25 and manually refined.

### 2.6. Atlas-Based Region-Specific Analysis

In order to assess the feasibility of the proposed approach for atlas-based region-specific analysis studies, we performed a comparison of term and preterm cohorts. The analysis was based on both the WM and γ^*av*^ parcellation maps. At first, all subjects (selected 40 preterm and 140 term subjects scanned between 38 and 43 weeks PMA range) were registered to the PMA-matched atlas space (section 2.3) with T2w, ODF, cortex and ventricle mask channels. It was identified experimentally, that adding the ventricle mask channel improves registration results for preterm subjects since preterm brains commonly have enlarged ventricles. Therefore, it was used for all subjects in the term-preterm comparison study.

The comparison analysis between the cohorts was performed in the atlas space. The structural and dMRI metrics were computed for each of the ROIs using robust weighted averaging with only the values with the difference <1.5 standard deviations from the mean included. The robust averaging helps to avoid errors due to image artifacts or local misregistration at the structure boundaries. The associations between the extracted metrics and the PMA at scan and the GA at birth were assessed using the standard ANOVA linear model analysis. The output *p*-values were corrected for multiple comparisons using the Bonferroni correction.

### 2.7. Implementation Details

The atlas was constructed with isotropic resolution 0.75*mm*. The LAC metric for MC registration of ODF channels was implemented in MRtrix3 (Tournier et al., 2019). In addition, we implemented the LNCC Demons metric (Avants et al., 2008) in MRtrix3 for registration of the structural channels which, although described in Raffelt et al. (2011), was not available in the current implementation of MRtrix3. We chose the default MRtrix3 registration parameters^8^ for multi-resolution ({0.5;0.75;1.0}), SH order (*l*_*max*_ = {0;2;2}), regularization of the gradient update field with Gaussian smoothing with 1 voxel standard deviation and regularization of the displacement field with Gaussian smoothing with 0.75 voxel standard deviation. For LNCC and LAC we chose the local neighborhood with 3 voxel radius (similarly to Raffelt et al., 2011). The proposed 4D GF fitting step (10) was implemented in MRtrix3. The ANOVA analysis for comparison between the term and preterm subjects was performed in RStudio (RStudio Team, 2020) using the standard lm() function.

## 3. Results and Discussion

### 3.1. Multi-Channel Registration

In our previous work (Uus et al., 2020) we have demonstrated that the proposed MC registration improves overall alignment of cortical and WM regions when driven by both structural and ODF channels in longitudinal cases. Here we confirm these results in cross-sectional registration. Additionally, we demonstrate that including the cortex mask as an additional channel improves accuracy of cortical alignment, which is otherwise decreased in the presence of ODF channel. This approach was also used in Makropoulos et al. (2018) and O'Muircheartaigh et al. (2020) to improve single-channel T2w registration.

**Figure 4:**
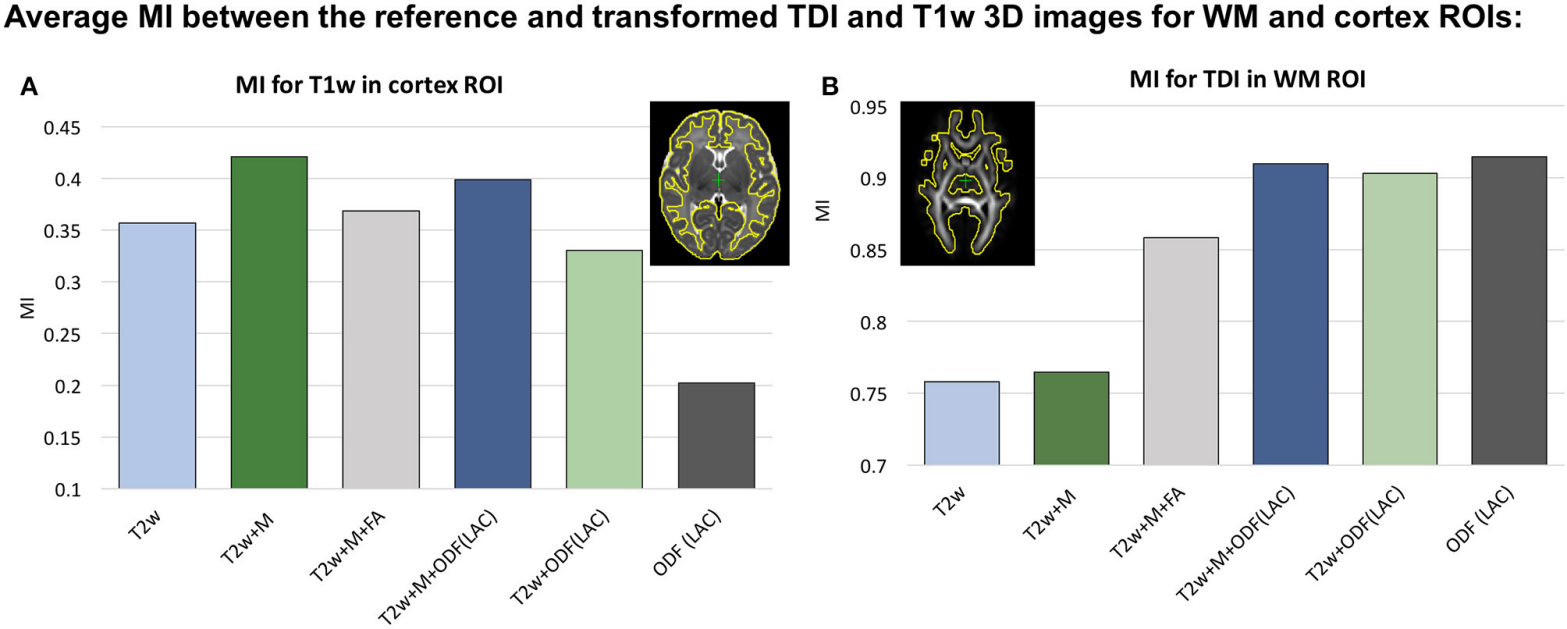
Comparison of MC registration results for different combinations of channels. The performance was measured by mutual information (MI) between aligned images and the age- and contrast-mached templates for **(A)** the T1w images in the cortical region and **(B)** TDI maps in the WM region. The regions are highlighted in yellow contours. The results are statistically significant with *p* < 0.001 for all cases apart from: all *ODF*-guided scenarios for the WM ROI, *T*2*w* vs. *T*2*w*+*M*_*cortex*_ for WM ROI and *T*2*w* vs. *T*2*w*+*M*_*cortex*_+*FA* for the cortex ROI.

We investigated six scenarios of registration of individual dHCP subjects to the templates $Y_{c}^{ref}\left(t\right)$ based on different combinations of channels: (I) *T*2*w*, (II) *T*2*w*+*M*_*cortex*_, (III) *T*2*w*+*M*_*cortex*_+*FA*, (IV) *T*2*w*+*M*_*cortex*_+*ODF*(*LAC*), (V) *T*2*w*+*ODF*(*LAC*) and (VI) *ODF*(*LAC*). The performance was tested on 11 term datasets from 42.00 to 42.57 weeks PMA since at this age the subjects have significantly higher degree of gyrification than the average templates. To assess the alignment in both WM and cortical regions we evaluated similarity of aligned individual images with the age- and contrast-matched templates using mutual information (MI) for (A) T1w channel in the cortical region and (B) TDI channel in the dilated WM region (highlighted in yellow in Figure 4). The mutual information similarity metric and the T1w and TDI channels were selected for evaluation to minimize bias toward the channels and similarity metrics used in registration.

We observed that all ODF-guided scenarios led to highest quality alignment of TDI maps (*p* < 0.001) and adding additional channels did not decrease the similarity after alignment (*p*>0.05). Including the FA channel improved TDI similarity compared to T2w and T2w+M (*p* < 0.001), but it was still significantly lower than for ODF guided alignments (*p* < 0.001) due to the contrast of poorly defined cortical features in FA. In the cortical region similarity of T1w contrast for the proposed *T*2*w*+*M*_*cortex*_+*ODF* MC registration was only slightly lower than the *T*2*w*+*M*_*cortex*_, but it was significantly higher than all the other scenarios (*p* < 0.001). Addition of the *M*_*cortex*_ channel improved the cortical alignment in all cases thus resolving the limitation reported in our previous work (Uus et al., 2020).

### 3.2. 4D Multi-Channel Atlas of Normative Neonatal Brain Development

**Figure 5:**
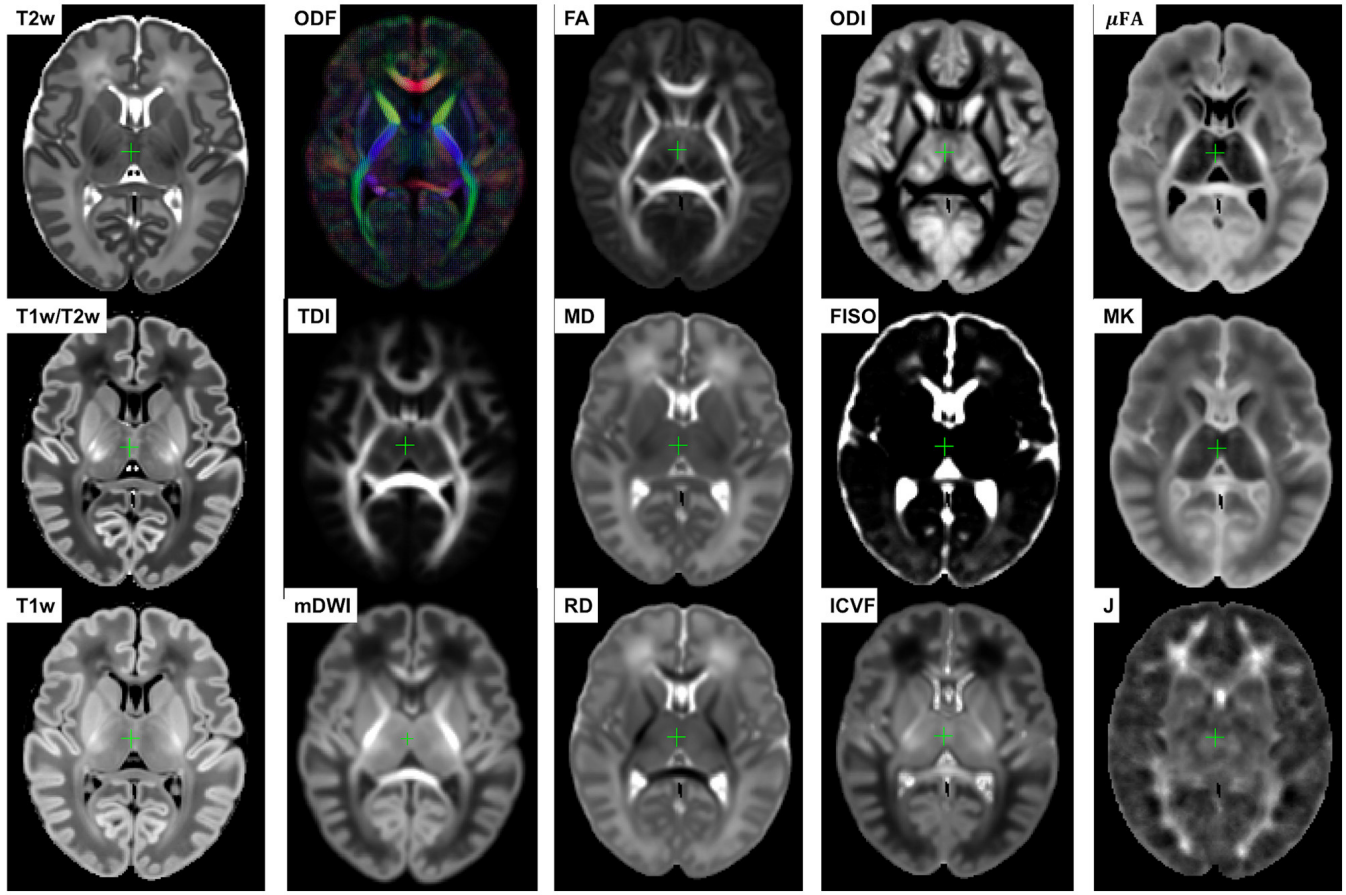
Multi-channel 4D atlas in the reference space (corresponding to 36 weeks PMA). Structural channels: T1, T2, T1/T2 and Jacobian; ODF channels: SH ODF, mDWI, TDI; DTI channels: MD, RD, FA; DKI channel: MK; NODDI channels: ODI, FISO, ICVF; μFA.

**Figure 6:**
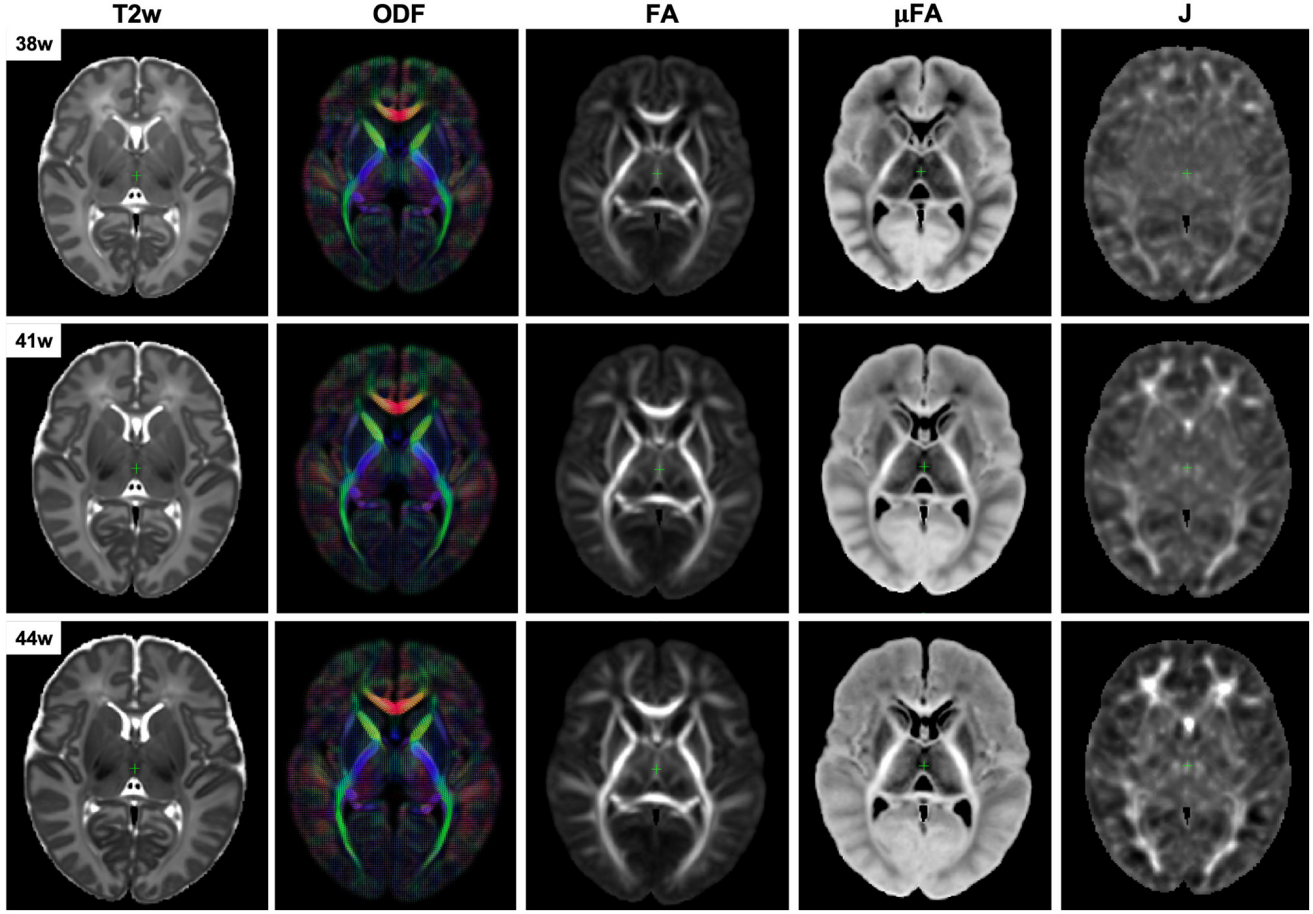
Example unbiased 4D atlas channels at 38, 41, and 44 weeks PMA. The corresponding Jacobian maps (J) are shown in the reference space.

The resulting multi-channel 4D atlas $Y_{c}^{ref}\left(t\right)$ in the reference space (36 weeks PMA dHCP atlas Schuh et al., 2018) is shown in Figure 5. Unbiased atlases *Y*_*c*_(*t*) obtained after application of average inverse warps for 38, 41 and 44 weeks PMA time points are presented in Figure 6. There are distinct nonlinear changes due to cortical folding in the T2w templates and volumetric expansion/contraction due to growth the is visible in the Jacobian maps.

**Figure 7:**
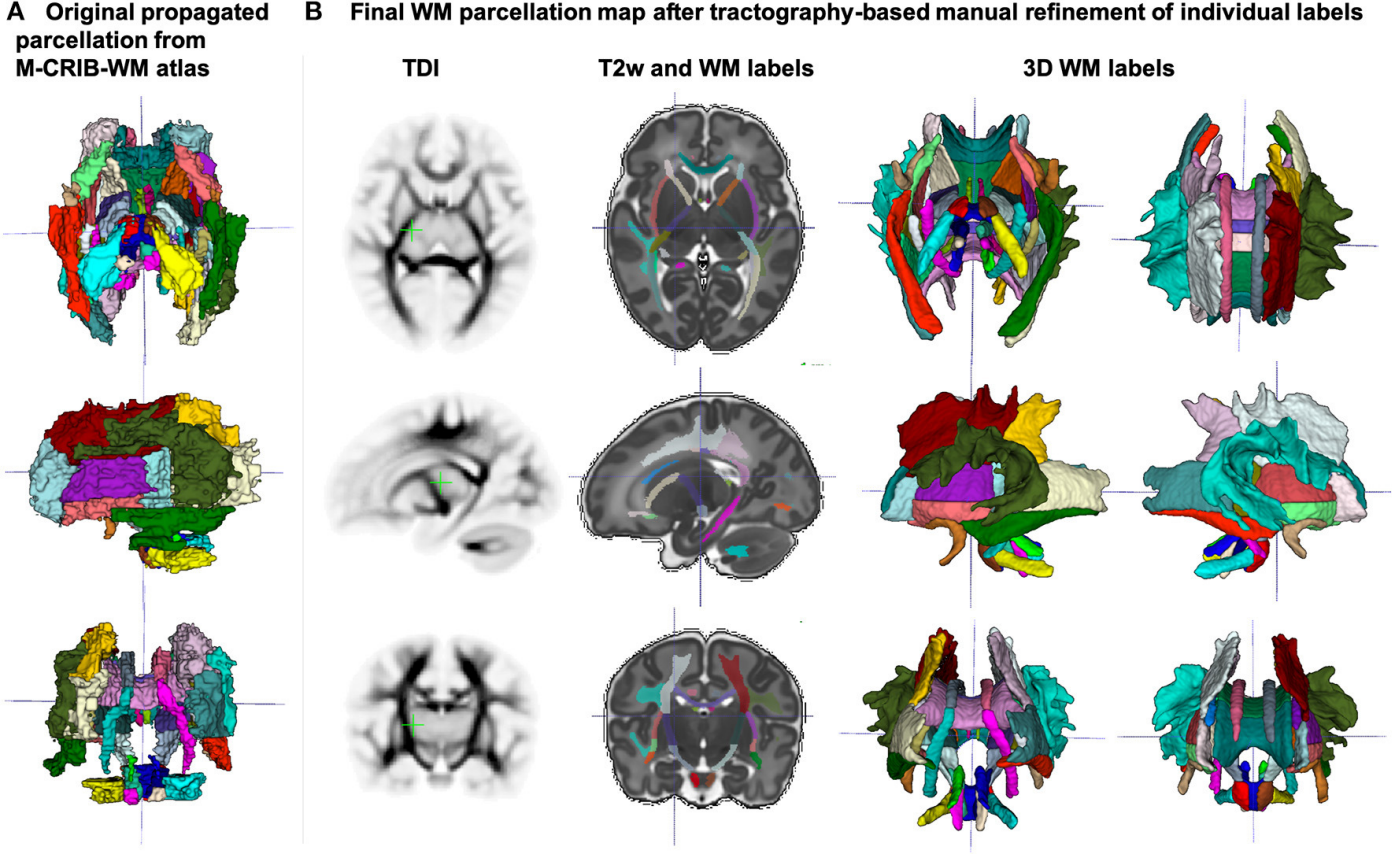
**(A)** Original WM parcellation map propagated from the M-CRIB-WM atlas using T2w-guided registration. **(B)** Final WM parcellation map after tractography-based manual refinement in the atlas reference space. The 54 ROIs are based on the structures defined in the M-CRIB-WM atlas (Alexander et al., 2020). The corresponding TDI map highlights the WM pathway regions.

The created WM parcellations map with 54 ROIs created in the atlas reference space (section 2.5) for the region-specific analysis of the metric values is shown in Figure 7B. The label annotation information follows the original annotations defined in Alexander et al. (2020). The tractography-based manual refinement of the originally propagated 2D-slice-wise segmentations (Figure 7A) from the M-CRIB-WM atlas provided a more accurate 3D definition of the WM ROIs that are developed by 44 weeks PMA. Furthermore, it removed the structural inconsistencies in the original 2D slice-wise WM segmentations that were performed on DTI directionally-encoded color maps.

**Figure 8:**
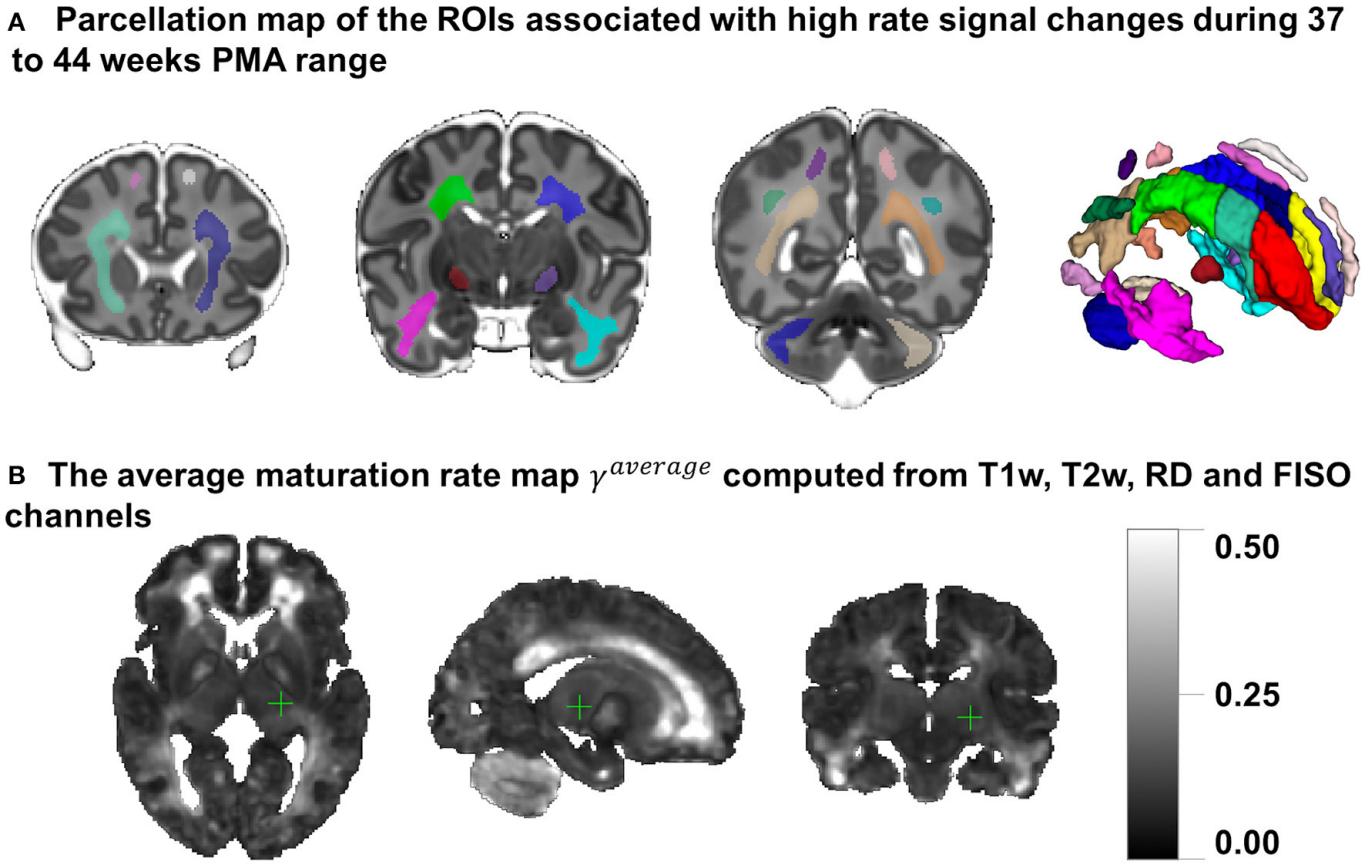
**(A)** The parcellation map of 24 paired regions identified by high change rates during 37–44 week PMA. **(B)** The average maturation rate map γ^*av*^ computed from T1w, T2w, RD, and FISO channels.

Figure 8A presents the parcellation map of the transient regions identified by high rates of signal changes during 37–44 weeks PMA segmented from the average γ^*av*^ map (Figure 8B). The parcellation map has 24 left/right regions with the majority being consistent with the transient fetal compartment regions described in the recently introduced extended MRI scoring systems of neonatal brain maturation (Pittet et al., 2019) including periventricular crossroads (Judaš et al., 2005), Von Monakow WM segments and subplate. We also identified fast developing regions within the cerebellum and subcortical gray matter.

**Figure 9:**
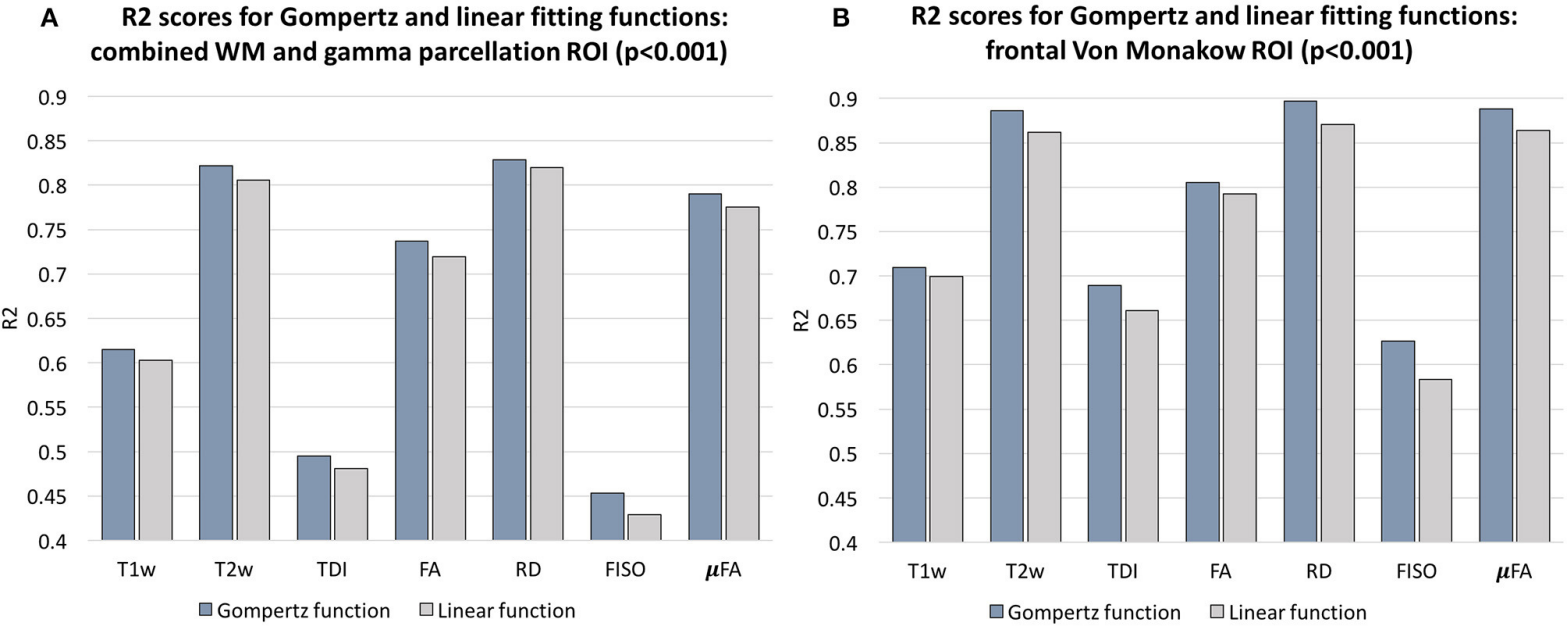
Comparison of the Gompertz function and linear fitting results in terms of *R*^2^ values evaluated within the combined WM and γ^*av*^ parcellation map regions **(A)** and frontal Von Monakow WM regions **(B)**. The results are statistically significant with *p* < 0.001.

In addition, we calculated voxel-wise *R*^2^ scores to evaluate the Gompertz function fit. Our results confirmed that GF offers higher *R*^2^ scores than linear regression with *p* < 0.001 for the combined γ and WM parcellation map region. The primary regions where the GF fitting outperformed linear fitting were the γ^*av*^ parcellation map and the local WM regions such as the frontal Von Monakow WM regions (labels 1 and 4 in the γ^*av*^ parcellation map). Figure 9 shows *R*^2^ values for GF vs. linear fitting comparison for a subset of channels.

**Figure 10:**
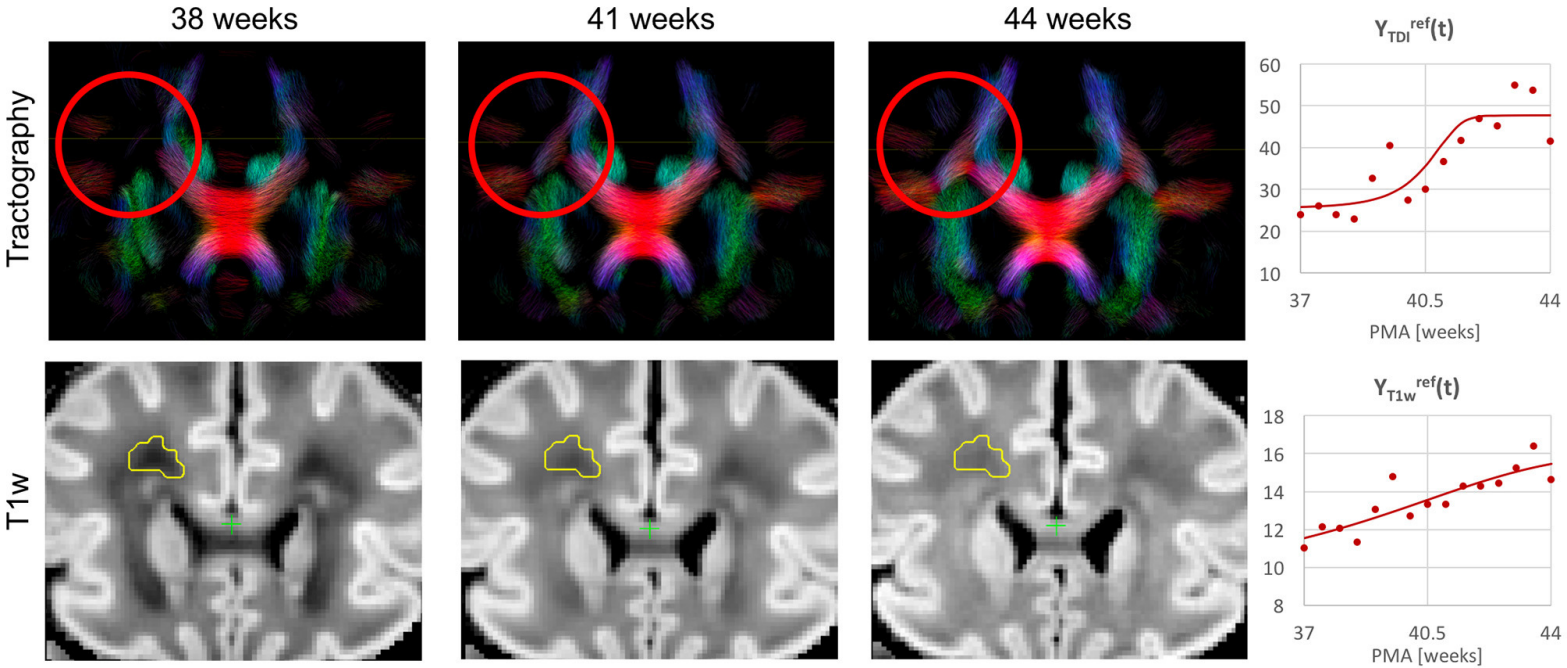
Whole brain probabilistic tractography generated from the ODF channel $Y_{ODF}^{ref}\left(t\right)$ and the corresponding T1w channel $Y_{T1w}^{ref}\left(t\right)$ (in the reference space) in the frontal WM region at 38, 41, and 44 weeks PMA time points. The developing WM pathway (red circle) can be linked to the increasing T1w signal intensity (yellow region). The graphs show the signal in age-specific templates $Y_{c,t}^{\left(2\right)}$ and fitted Gompertz function $Y_{c}^{ref}\left(t\right)$ in the TDI and T1w channels averaged over the region highlighted in yellow.

**Figure 11:**
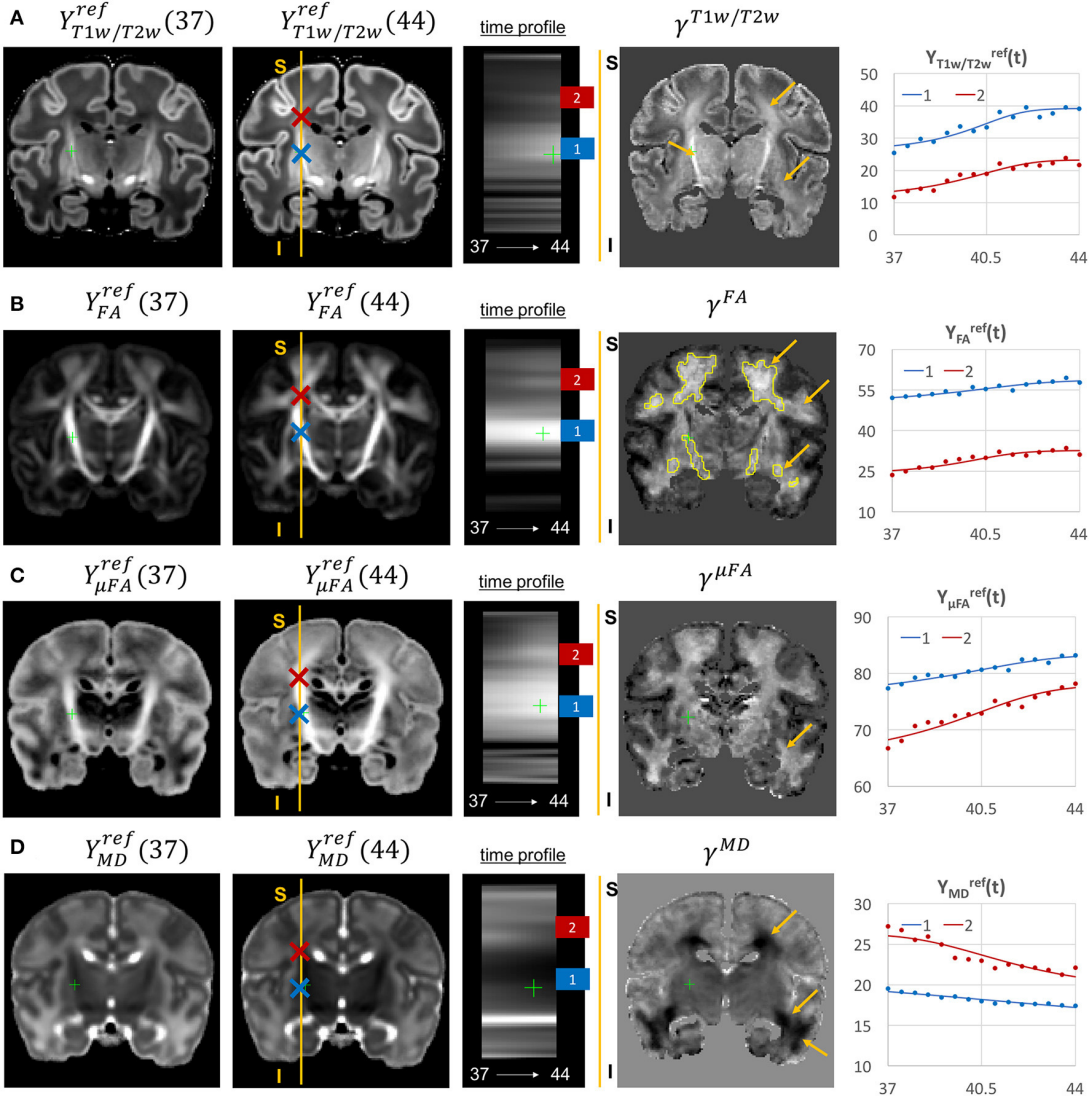
Examples of the signal changes in time (in the reference space) in T1w/T2w **(A)**, FA **(B)**, μFA **(C)**, and MD **(D)** channels. First column: 37 week template. Second column: 44 week template. Third column: signal change in time. Fourth column: γ^*c*^ maps. Fifth column: Signal change in time in age-specific templates $Y_{c,t}^{\left(2\right)}$ and fitted Gompertz function $Y_{c}^{ref}\left(t\right)$ computed over 3 × 3 × 3 voxel regions in two locations: PLIC (blue) and superior corona radiata (red). The regions highlighted with yellow contours have >0.2 weeks growth peak offset in τ^*c*^.

**Figure 12:**
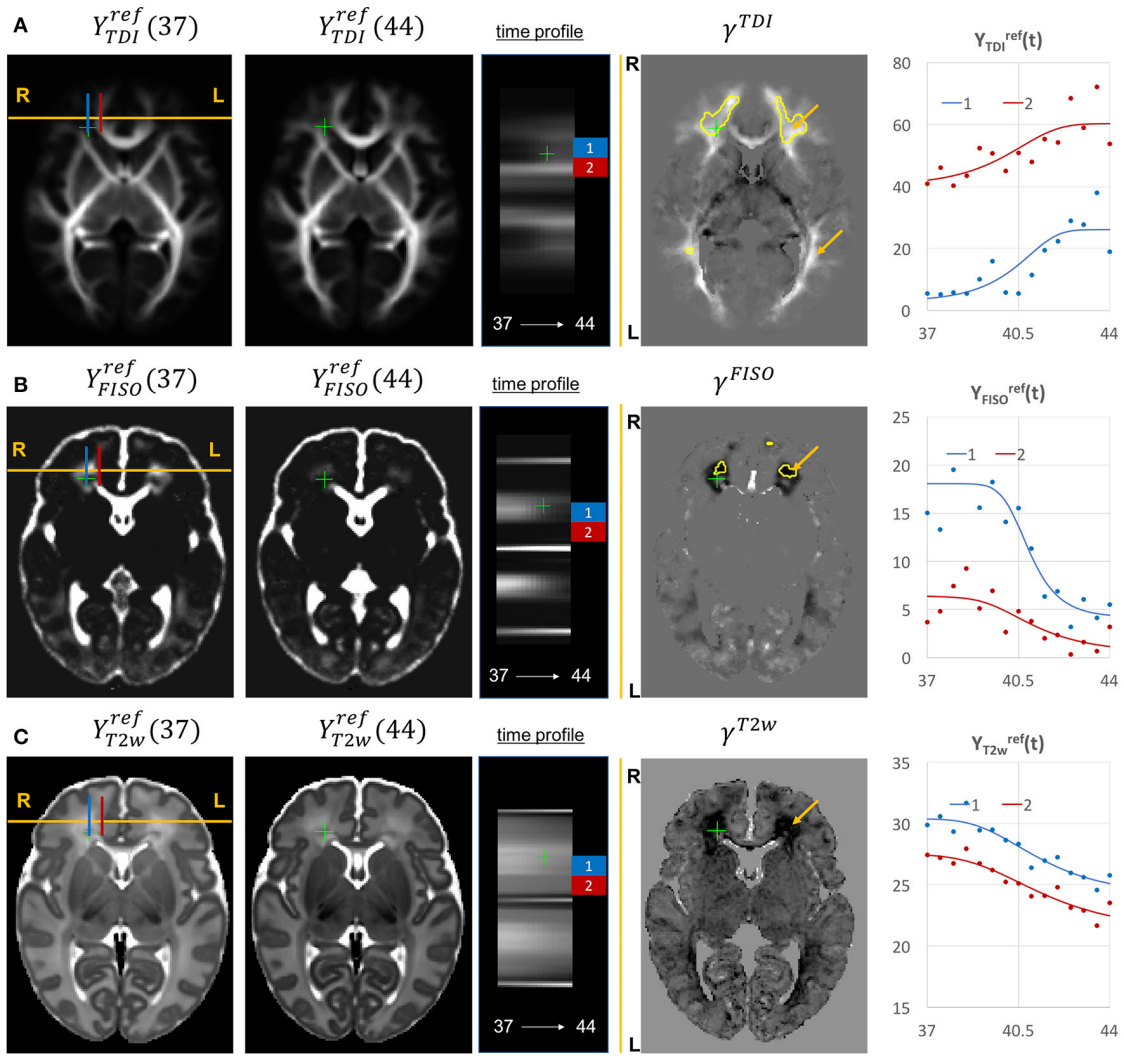
Examples of the signal changes in time (in the reference space) in TDI **(A)**, FISO **(B)**, and T2w **(C)** channels. First column: 37 week template. Second column: 44 week template. Third column: signal change in time. Fourth column: γ^*c*^ maps. Fifth column: Signal change in time in age-specific templates $Y_{c,t}^{\left(2\right)}$ and fitted Gompertz function $Y_{c}^{ref}\left(t\right)$ computed over 3 × 3 × 3 voxel regions in two locations: prefrontal corpus callosum (red) and Von Monakow WM segment (blue). The regions highlighted with yellow contours have >0.2 weeks growth peak offset in τ^*c*^.

**Figure 13:**
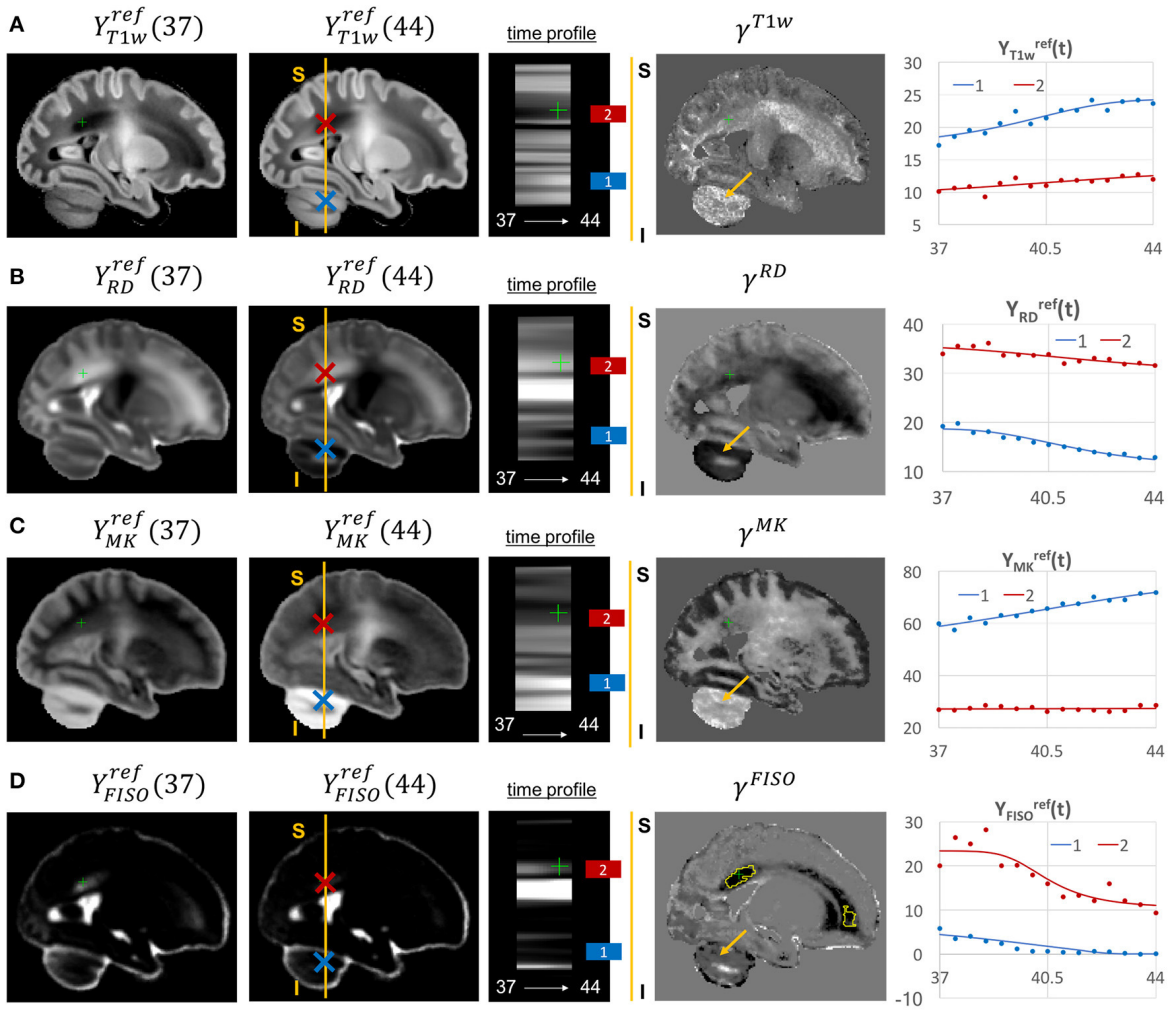
Examples of the signal changes in time (in the reference space) in T1w **(A)**, RD **(B)**, MK **(C)**, and FISO **(D)** channels. First column: 37 week template. Second column: 44 week template. Third column: signal change in time. Fourth column: γ^*c*^ maps. Fifth column: Signal change in time in age-specific templates $Y_{c,t}^{\left(2\right)}$ and fitted Gompertz function $Y_{c}^{ref}\left(t\right)$ computed over 3 × 3 × 3 voxel regions in two locations: cerebellum (blue) and periventricular crossroads (red). The regions highlighted with yellow contours have >0.2 weeks growth peak offset in τ^*c*^.

Examples of the non-linear patterns in signal changes also can be observed in the graphs in Figures 10–**13** showing average signal values in 3 × 3 × 3 voxel ROIs and the corresponding average GF fitting results. However, the relatively small improvement in *R*^2^ suggests that a linear fit also offers a reasonable approximation during this short time-window and that it is acceptable to use the linear model based ANOVA analysis for interpretations of trends in early neonatal brain development.

### 3.3. Visual Analysis of Normal Neonatal Brain Development

Figure 10 shows the output of iFOD2 probabilistic tractography (Tournier et al., 2010) generated from the ODF channel and the corresponding T1w channel (in the reference space) in the frontal WM region at 38, 41, and 44 weeks PMA time points. The increase in the T1w signal (known to be sensitive to proliferation of cells and myelin precursors and decreasing water content Girard et al., 2012) can be linked to the developing WM pathways seen in tractography (highlighted in red circle). The graphs show the corresponding increasing intensities in the age-specific average templates $Y_{c,t}^{\left(2\right)}$ and fitted signal values $Y_{c}^{ref}\left(t\right)$ of the TDI and T1w channels computed in the small frontal Von Monakow WM segment (Pittet et al., 2019) highlighted in yellow in the T1w channel.

The examples of signal intensity changes in time in different channels and the corresponding growth rate maps γ^*c*^ are presented in Figures 11–**13**. The regions highlighted in yellow have a growth peak offset in time ≥ 0.2 weeks from the 40.5 weeks central time point in τ^*c*^ and can be interpreted as indicators of earlier or later maturation with respect to the central time point of 40.5 weeks PMA. The graphs show average signal values in 15 discrete age-specific templates $Y_{c,t}^{\left(2\right)}$ and the corresponding fitted signal $Y_{c}^{ref}\left(t\right)$ calculated within small 3 × 3 × 3 voxel regions at specific locations, including the right posterior limb of internal capsule (PLIC), superior corona radiata, periventricular crossroads, corpus callosum, Von Monakow WM segment and cerebellum.

The WM tracts are characterized by different maturation times and rates (Iida et al., 1995). The T1w/T2w contrast (linked to myelination by Glasser and Van Essen, 2011) shows gradual signal increase from 37 to 44 weeks (Figure 11A). The γ^*T*1*w*/*T*2*w*^ map and the average signal graphs *Y*_*T*1*w*/*T*2*w*_(*t*) confirm that the rate of T1w/T2w signal increase is the highest in the PLIC region (blue) and the corona radiata (red). The value of the τ^*T*1*w*/*T*2*w*^ parameter of the Gompertz function is approximately 40.5 weeks in both regions which is in agreement with the previously reported myelination milestones (Counsell et al., 2002; Wang et al., 2019). There is also a noticeable increase in the cortical T1w/T2w signal, also previously reported by Bozek et al. (2018), which may be due to the ongoing myelination or the increased cell density (Girard et al., 2012). Both FA and μFA signals (Figures 11B,C) gradually increase in all WM regions in agreement with the trends reported in Feng et al. (2019) and Dimitrova et al. (2020). The μFA map shows generally higher degree of changes than FA, potentially due to the increasing crossing fiber effect, while in γ^*FA*^, the more prominent WM changes are observable primarily in the corona radiata, sagittal stratum and superior longitudinal fasciculus as well as the parietal crossroads and subplate (highlighted with arrows). The γ^*MD*^ map of the MD channel (Figure 11D) shows a large decrease in the superior corona radiata, sagittal stratum and the transient fetal compartments associated with WM maturation (Judaš et al., 2005; Pittet et al., 2019) including the periventricular crossroads and subplate regions (highlighted with arrows). The MD signal is slowly decreasing the PLIC region as can be seen in the corresponding graph (blue). All of the presented γ^*c*^ maps also show significant changes in the periventricular parietal crossroad regions (highlighted with arrows) with the significant decrease in MD and increasing in T1w/T2w.

Given the fixed number of streamlines used for probabilistic tractography, there is a notable redistribution of the TDI amplitude from the main to proximal WM tracts (Figure 12A). The corresponding growth rate γ^*TDI*^ map is positive in the frontal (anterior corona radiata) and thalamic radiation WM regions (highlighted with arrows) and negative in the internal capsule. The R-L time profile in the frontal region (Von Monakow WM segment, blue) shows the increased track density at 44 weeks. The average TDI signals $Y_{TDI}^{ref}\left(t\right)$ in this region (blue) and the corpus callosum (red) are also characterized by a significant degree of nonlinearity. the NODDI FISO component (Figure 12B) shows a prominent reduction in the same frontal region which is in agreement with the expected decrease of water content and progressing maturation of WM pathways (Girard et al., 2012). Similarly to TDI, the average FISO signals $Y_{FISO}^{ref}\left(t\right)$ in the investigated WM ROIs have nonlinear shape with the steep decrease occurring during the 39.5–43 weeks period. A similar decrease is observed in T2w signal (Figure 12C). The FISO channel in the sagittal view in Figure 13D also demonstrates similar patterns in the periventricular crossroads (red).

Most of the channels also show prominent changes in the cerebellum associated with the normal maturation process (Figure 13, blue). The T1w signal intensity $Y_{T1w}^{ref}\left(t\right)$ is gradually increasing due to WM development along with the increasing microstructural complexity reflected in the MK channel with the high γ^*MK*^ map values and the expected decreasing trends of the RD $Y_{RD}^{ref}\left(t\right)$ and FISO *Y*_*FISO*_(*t*) signals (potentially due to the decreasing amount of free water Girard et al., 2012).

### 3.4. Atlas-Based Region-Specific Analysis

In order to demonstrate the feasibility of the proposed MC atlas-based analysis approach and give an example of one of the possible applications, we performed ANOVA analysis to assess the influence of GA at birth on microstructure of WM regions delineated in our new atlas, with PMA as a confounding variable. To assess the feasibility of using the ANOVA analysis for the investigated datasets, we performed linear fitting for each of the channels. The γ^*c*^ values showed high correlation with the linear slope maps with the average NCC for all channels in the whole brain ROI 0.90∓0.09 (without CSF).

This is in agreement with the appearance of the global trends in Figures 10–13 as well as the other reported studies (Feng et al., 2019; Dimitrova et al., 2020; O'Muircheartaigh et al., 2020) and confirms that during the short period between 37 and 44 weeks PMA range a linear approximation can be considered to be acceptable for ANOVA-based studies.

**Figure 14:**
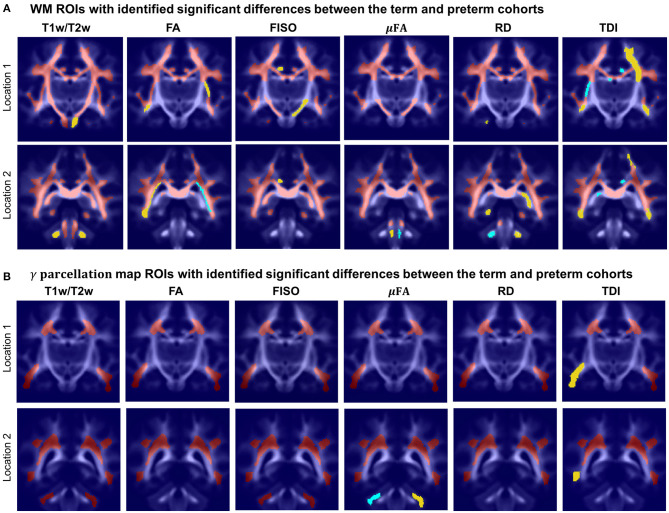
Atlas-based region-specific analysis. The regions significantly associated with GA at birth are highlighted with red (*p* < 0.001), yellow (*p* < 0.01) and cyan (*p* < 0.05) and overlaid over the averaged TDI map in two coronal view locations. **(A)** WM parcellation regions. **(B)** γ^*av*^ parcellation regions.

Figure 14 visualizes WM and transient regions in selected channels where average signal value was significantly associated with GA at birth. The main regions that have significant correlation of multiple indices with GA include: the corona radiata, superior longitudinal fasciculus, corpus callosum and thalamic radiation. The T1w/T2w contrast also showed to have significant correlation with GA in the internal and external capsule ROIs (Figure 14A). There is also a significant difference between the cohorts within the majority of γ^*av*^ parcellation regions (Figure 14B), which is in agreement with the expected prolonged existence of transient compartments in preterm subjects (Kostović and Judaš, 2006).

**Figure 15:**
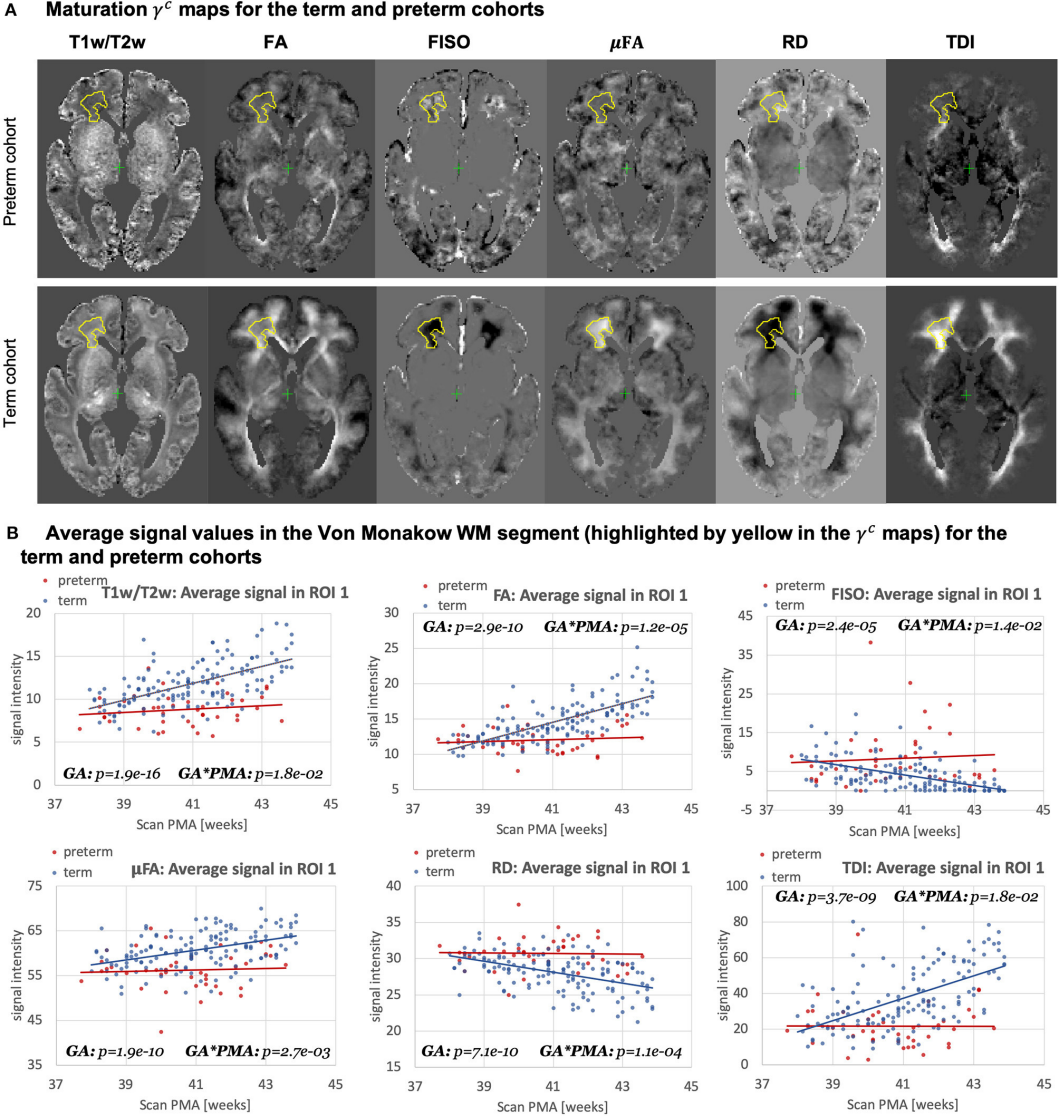
Atlas-based analysis: comparison of the term (140) and preterm cohorts (40) for 38 to 43 weeks scan PMA range for a subset of channels c={ T1w/T2w; TDI; RD; FA; FISO; μFA }. **(A)** The γ^*c*^ maps of GF fitting for the term and preterm cohorts for 38 to 43 weeks PMA range. **(B)** The mean signal values in the frontal WM ROI from the γ^*average*^ parcellation map (highlighted in yellow in the gamma maps) for the term (blue) and preterm (red) cohorts for 38–43 weeks PMA range.

Figure 15A highlights the differences in the maturation rate γ^*c*^ maps between the term and preterm cohorts. The graphs in Figure 15B show the average signal values in the frontal right Von Monakow WM segment (highlighted in yellow in the γ^*c*^ maps). The rather wide range of values in all indices is potentially related to both the large size of the investigated WM region (approximately 3000 voxels) as well the individual variability also commonly observed in other neonatal brain studies (Feng et al., 2019; O'Muircheartaigh et al., 2020). There is a clear increasing trend in T1w/T2w, FA and TDI for the term cohort along with decreasing FISO and RD. However, the slopes for the preterm cohort are close to zero with high variance in the signal values. Furthermore, in this region, the preterm subjects are characterized by significantly higher FISO and RD values and lower T1w/T2w, TDI and FA than the term cohort at the 42–43 week PMA period. This is consistent with the commonly reported lower FA and higher diffusivity values in preterm groups (Hermoye et al., 2006; Knight et al., 2018; Dimitrova et al., 2020), again suggesting delayed maturation of transient compartments in premature babies (Kostović and Judaš, 2006).

## 4. Limitations and Future Work

The generated atlas is specific to the dHCP acquisition protocols, which might limit its application in terms of comparison with datasets from other studies. However, the proposed tools can be applied to generate study- and acquisition-specific 4D MC atlases. We investigated a relatively narrow neonatal period, and extension to a wider age range would improve the reliability of the Gompertz function fit and bring more insights into early brain development. In addition, a detailed region-specific statistical evaluation of the expected signal distributions of MRI-derived indices within the normal term cohort would need to be performed to allow accurate detection of image artifacts and brain abnormalities. Furthermore, the current work did not investigate the optimal preprocessing parameters required for fitting NODDI and μ*FA* dMRI models, the effect of filtering (e.g., Smith et al., 2015) on the tractography outputs or the impact of different registration settings (e.g., channel weighting).

The study comparing term and preterm brain development included only 40 preterm subjects and they were not grouped with respect to specific types of anomalies, which can be addressed in future as more datasets become available. Furthermore, this work did not evaluate the influence of multi-channel registration on the extracted values of different microstructural indices. The generated WM parcellation map also potentially requires additional verification with respect to the correct definition of individual WM regions. Including additional cortical and sub-cortical regions or fixel-based analysis (Raffelt et al., 2017) could also enrich the insights into normal and preterm microstructural brain development.

## 5. Conclusions

In this work, we proposed and implemented a novel pipeline for generation of continuous 4D multi-channel atlases. It is based on multi-channel ODF+T2w+M_*cortex*_ guided registration and the Gompertz function fitting of both signal intensities and spatial transformations. The multi-channel registration pipeline implemented in MRtrix3 employs the novel local angular correlation similarity metric for ODF channels, LNCC metric for structural T2w and weighted fusion of the updates to the displacement fields. It also includes the cortex mask channel guided by LNCC metric for better alignment of the cortical regions.

Based on the proposed methods, we generated the first continuous multi-channel atlas of the normal term neonatal brain development during 37–44 weeks PMA generated from 170 subjects from the dHCP project. The atlas contains 14 channels including structural (T1w, T2w and T1w/T2w contrast) and DWI-derived metrics based on ODF, DTI, DKI, μFA and NODDI models. The Gompertz function fitting of the signal intensity and spatial transformation components in 4D allowed parametrization of the atlas. The output γ maps representing the rate of change can be used for interpretation of how maturation processes are manifested in different structural and diffusion MRI-derived metrics. Visual inspection of the fitting results showed that γ^*c*^ maps of the T2w, T1w, FISO, MD, RD and TDI channels are characterized by the high contrast in the fetal transient compartments (Pittet et al., 2019).

The atlas also includes two detailed WM parcellation maps: (i) the map with the major WM tract ROIs based on the definitions from the recently introduced M-CRIB-WM neonatal atlas (Alexander et al., 2020) and (ii) the map of the regions associated with high γ signal change rates during the normal WM maturation process. We tested the applicability of these parcellation maps for region-specific atlas-based studies on comparisons between the term and preterm cohorts. The results of this study showed significant effects linked to prematurity in multiple WM regions including the transient fetal compartments. The atlas and the software tools will be publicly available after publication of the article to support future studies of early brain development^1^.

In summary, the proposed multi-channel registration and atlas facilitate combined analysis of structural and diffusion MRI indices in the same reference space without a bias from single-channel registration. Furthermore, combination of high resolution T2w and cortex mask channels with low resolution ODF channels aids better combined alignment of cortical and WM structures. To our knowledge, this is the first work that defines the pipeline for merged structural and diffusion MRI atlas-based analysis in neonatal brain studies.

## Data Availability Statement

The data analyzed in this study is subject to the following licenses/restrictions. The neonatal MRI datasets will be available in the dHCP project data release. Requests to access these datasets should be directed to http://www.developingconnectome.org/second-data-release.

## Ethics Statement

The studies involving human participants were reviewed and approved by London - Riverside Research Ethics Committee. Written informed consent to participate in this study was provided by the participants' legal guardian/next of kin.

## Author Contributions

AU prepared the manuscript, implemented the code for the extended MC registration, fitting and analysis, generated the 4D MC atlas and conducted the experiments. IG participated in implementation of the preprocessing and analysis code, the design of the study and interpretation of the results. MP developed the original code for SSD MC ODF registration in MRtrix3. DB performed preprocessing of the dHCP datasets. MP, DC, and J-DT developed the tools for preprocessing and analysis of HARDI dHCP datasets. JH, EH, J-DT, LC, and AP developed MRI acquisition protocols for the neonatal dHCP datasets. LC developed the tools for preprocessing of structural dHCP datasets. JVH, ADE, SC, and MR are coordinators of the dHCP project. MD conceptualized the study and the methods, obtained the funding and supervised all stages of the research and preparation of the manuscript. All authors gave final approval for publication and agree to be held accountable for the work performed therein.

## Conflict of Interest

The authors declare that the research was conducted in the absence of any commercial or financial relationships that could be construed as a potential conflict of interest.

## Footnotes

^1^4D MC neonatal brain atlas: https://gin.g-node.org/alenaullauus/4d_multi-channel_neonatal_brain_mri_atlas

^2^dHCP project: http://www.developingconnectome.org.

^3^MRtrix3 toolbox: https://www.mrtrix.org.

^4^FSL toolbox: https://fsl.fmrib.ox.ac.uk.

^5^ANTs toolbox: http://stnava.github.io/ANTs.

^6^DRAW-Em toolbox: https://github.com/MIRTK/DrawEM.

^7^dHCP weekly neonatal brain atlas: https://gin.g-node.org/BioMedIA/dhcp-volumetric-atlas-groupwise.

^8^MRtrix3 mrregister function: https://mrtrix.readthedocs.io/en/latest/reference/commands/mrregister.html.
